# Rho-associated kinase (ROCK)-associated proteins and genes-encoded proteins upregulated in lung squamous cell carcinoma (LUSC)

**DOI:** 10.1186/s43046-026-00351-0

**Published:** 2026-04-20

**Authors:** Muhammad Asyaari Zakaria, Nor Fadilah Rajab, Eng Wee Chua, Muhammad Redha Abdullah Zawawi, Siti Fathiah Masre

**Affiliations:** 1 Centre for Toxicology and Health Risk Studies, Faculty of Health Sciences, Universiti Kebangsaan Malaysia, Kuala Lumpur, 50300 Malaysia; 2 Centre for Healthy Ageing and Wellness, Faculty of Health Sciences, Universiti Kebangsaan Malaysia, Kuala Lumpur, 50300 Malaysia; 3 Faculty of Pharmacy, Universiti Kebangsaan Malaysia, Kuala Lumpur, 50300 Malaysia; 4 UKM Medical Molecular Biology Institute (UMBI), Universiti Kebangsaan Malaysia, Jalan Yaacob Latif, Kuala Lumpur, Cheras 56000 Malaysia; 5 Faculty of Pharmacy and Health Sciences, Royal College of Medicine Perak, University of Kuala Lumpur, Ipoh, Perak 30450 Malaysia

**Keywords:** Lung squamous cell carcinoma (LUSC), Carcinogenesis, Pre-malignant, Malignant, N-nitroso-tris-chloroethylurea (NTCU), Rho-associated kinase (ROCK), Differentially expressed genes (DEGs), Protein-protein interaction (PPI)

## Abstract

**Background:**

The Rho-associated kinase (ROCK) signaling pathway was reported to be overexpressed in cancer, as it promotes multiple hallmarks of cancer. However, the characterization of ROCK signaling pathway expression in dual-stage carcinogenesis of lung squamous cell carcinoma (LUSC) remains unclear. Therefore, this study aims to evaluate ROCK signaling pathway expression during LUSC progression *in vivo*.

**Methods:**

Female BALB/c mice were treated with N-nitroso-tris-chloroethylurea (NTCU) to induce pre-malignant and malignant LUSC stages. The lung tissues were subjected to immunohistochemistry and Western blot for ROCK-associated protein expression analysis. Then, differentially expressed genes (DEGs) analysis from two expression profiling datasets: GSE30219 and GSE31446, was performed to determine the mRNA expression of ROCK-associated proteins in human LUSC. Protein-protein interaction (PPI) network (analysed via Cytoscape) and correlation analysis (analysed via Gene Expression Profiling Interactive Analysis 2; GEPIA2) were also conducted to identify the relationships between each ROCK-associated protein.

**Results:**

This study found a significantly higher expression (p-value<0.05) of ROCK-associated proteins such as pFAK, RhoABC, ROCK1, ROCK2, and pMLC during LUSC progression via IHC and Western blot assays. Notably, RhoA, RhoB, and pMLC (MYL9) mRNA were significantly upregulated in human LUSC. The PPI and correlation analysis identified RhoA, ROCK1, and ROCK2 as highly correlated ROCK-associated genes encoded proteins in LUSC.

**Conclusion:**

Increased ROCK-associated proteins and genes expression in dual-stage carcinogenesis of LUSC may reflect their essential role in cancer growth. Bioinformatic analysis results verify the importance of ROCK signaling pathways in LUSC growth, making them promising targets for future LUSC treatment.

**Supplementary Information:**

The online version contains supplementary material available at 10.1186/s43046-026-00351-0.

## Introduction

The non-small cell lung cancer, or NSCLC, accounts for > 85% of all lung cancer cases. NSCLC is a heterogeneous disease categorized into three main subtypes: adenocarcinoma, lung squamous cell carcinoma (LUSC), and large cell carcinomas [1]. Over the past decade, new approaches to treat NSCLC have emerged, but few advances have been made in LUSC treatment, making it a subtype of priority for further research. Affecting around 400,000 people each year [2], LUSC is often diagnosed at a late stage due to a lack of screening modalities that can detect the disease early, especially in developing countries [3]. Moreover, it also yields a high mortality rate and is associated with a poor therapeutic response [2, 4]. Thus, research on the LUSC subtype is crucial to understand the disease better.

LUSC originates from basal-like stem cells, alveolar type 2 (AT2) cells, and club cells [5]. This subtype has more common characteristics with other squamous cell carcinomas (SCC) that arise from different anatomic locations than with lung adenocarcinoma [6]. The example of causative factors that can increase the probability of LUSC formation are smoking, alcohol consumption, and infections that induce long-term DNA damage, such as Epstein-Barr virus (EBV) and human papillomavirus (HPV) [7, 8]. LUSC presents with a challenging therapeutic response due to its complex genetic background, high mutation rate, and chromosomal instability [2, 9, 10]Thus, thorough understanding of the molecular mechanisms underlying LUSC progression is crucial in improving therapeutic strategies.

For the past decade, advances in molecular techniques have provided researchers with essential knowledge on the pathways responsible for lung cancer etiology. One of the pathways recognized to play a critical role in promoting lung cancer growth is Rho-associated kinase (ROCK) [11, 12]. ROCK is a family of serine/threonine kinases and consists of two isoforms, ROCK1 and ROCK2, which share 65% overall identity and 92% amino acid sequence similarity in the kinase domain [13]. ROCK plays a profound role in many physiologic functions of cells, including cell migration, adhesion, division, and death [14]. However, ROCK was reported to take part in several diseases including cancer, as its expression increased [15–18]. Mechanistically, ROCK was activated via phosphorylated-focal adhesion kinase (FAK) or pFAK, an active form of FAK that senses the increasing extracellular force due to rigid tissue (a common physical characteristic of cancer tissue). Increased rigidity or stiffness of cancer tissue is contributed by the buildup of hyperproliferative cancer cells, recruitment of stromal cells, and increased synthesis of extracellular matrix proteins [19]. For example, the rigidity of lung fibrosis which resembles lung cancer tissue has been reported to increase until 17 kPa from 2 kPa in normal lung tissue [20]. pFAK has been well-documented as a major upstream effector of multiple GTPase proteins such as Rac, Ras, and Rho [21, 22]. Among others, Rho GTPase has been suggested to be responsible for mediating cancer progression, including lung cancer [23]. Then, Rho GTPases (RhoA/B/C) will become active, which will bind to ROCK protein and change its conformation, enabling it to activate downstream substrates such as myosin light chain or MLC(MYL9), myosin phosphatase target or MYPT, and LIM kinase subunit 1/2 or LIMK1/2 [24–26]. Among other substrates, MLC(MYL9) or phosphorylated-MLC (pMLC), an active form of MLC is the main downstream substrate of ROCK1/2 responsible for inducing myosin crosslinking to filamentous actin (F-actin). This cross-linking results in cytoskeleton contractility, which is essential for regulating multiple cell behaviors [23]. Thus, pFAK is an important protein that mediates RhoABC-ROCK signaling pathway activation, which will eventually activate MLC to become pMLC. In cancer, the dysregulation of this cascade promotes cellular phenotypes that favor tumor growth, including increased cancer cell proliferation, migration, and metastasis [27].

ROCK1/2 expression varied across different cancer types [19, 28, 29]. Several studies have reported a positive association between ROCK activation and tumor growth [28, 30, 31], and high levels of expression are predominantly observed in late-stage cancer [29, 32]. However, most of the previous studies on lung cancer only evaluated the expression of ROCK1 isoform [32–35], over ROCK2, which is also reported to play a significant role in promoting multiple hallmarks of cancer [31, 36]. Moreover, most previous studies evaluating ROCK expression were only conducted in vitro, in adenocarcinoma [32, 37–40] and in large cell lung cancer subtypes [36, 39, 41, 42]. Briefly, they suggested that ROCK overexpression plays an essential role in promoting the growth of adenocarcinoma and large cell lung cancer subtypes. Therefore, determining the expression of ROCK signalling pathway activation in LUSC subtype is crucial. Investigation of ROCK1 and ROCK2 expression in dual-stage carcinogenesis (pre-malignant and malignant) of LUSC is necessary to elucidate the role of ROCK in LUSC progression in vivo, as performed in this study, using our established N-nitroso-tris-chloroethylurea (NTCU)-induced LUSC mouse model [43]. Additionally, mRNA expression analysis of ROCK-associated genes-encoded proteins in human LUSC, curated from public databases, may help to verify their profound roles in LUSC. These approaches may provide valuable insights for the development of a novel ROCK-targeted cancer treatment strategies.

LUSC was developed in the mouse model using NTCU, an SCC carcinogen, as performed in our previous work [43]. The utilization of chemical-induced tumors, such as NTCU, was suggested as the best technique to study carcinogenesis since it can mimic the chronic exposure of humans to lung cancer risk factors. This chronic exposure to NTCU in vivo enables the development of LUSC through a hyperplasia-squamous metaplasia-squamous dysplasia-SCC sequence, similarly observed in human LUSC [44–48]. Moreover, the mice were reported to harbor a genetic profile highly similar to human LUSC, as confirmed by Xiong et al. [49] and Riolobos et al. [50]. Following NTCU administration, the pre-malignant and malignant LUSC tissues were harvested and subjected to histological analysis. Then, the expressions of associated ROCK proteins; pFAK, RhoABC, ROCK1, ROCK2, and pMLC(MYL9) were determined via immunohistochemistry staining and Western blot to evaluate ROCK signaling pathway activation during LUSC carcinogenesis in vivo. We also investigated mRNA expression, protein-protein interactions, and correlations among ROCK-associated genes-encoded proteins in human LUSC to better understand ROCK involvement in this deadly disease.

## Materials and methods

### Animal

All animal handling procedures were approved by the Universiti Kebangsaan Malaysia Animal Ethics Committee (UKMAEC) (FSK/2017/FATHIAH/24-MAY/846-MAY-2017-MAY-2020) and in compliance with the ARRIVE guidelines. All methods were performed following the relevant guidelines and regulations. LUSC animal model development has been conducted in our previously published work [43]. 24 female BALB/C mice (five weeks) with an average weight of 12–16 g were purchased from the animal unit, Faculty of Veterinary Medicine, Universiti Putra Malaysia. The mice were acclimatized in the same animal house for 14 days with ad libitum access to mouse pellets and tap water. The ambient room temperature and lighting (12 h light-dark cycle) were also ensured in the animal house throughout the experiment.

### Development of lung cancer in mice

Mice were allotted into two main groups, Pre-malignant (PM) and Malignant (M), which received treatment for 15 and 30 weeks, respectively. Then, the mice in each group were further allotted into two groups (*n* = 6); Vehicle (receiving 70% acetone) and Cancer group (receiving 0.04 M NTCU). 25 µl of treatment was applied to the dorsal area, between the shoulder blade bones, on the shaved skin of mice via skin painting [47]. NTCU was administered twice per week (an interval of ~ 3.5 days) for 15 and 30 weeks to induce pre-malignant and malignant LUSC, respectively. At the end of 15 and 30 weeks after the initial treatment, the animals were terminated using an overdose of ketamine and xylazine (KTX), 0.2 ml/20 mg through intraperitoneal injection and followed by cervical dislocation. After termination, the lungs were harvested and subjected to histology assays to confirm the formation of the LUSC subtype. The lung tissues used in this study are from our published work [43].

### Immunohistochemistry staining

IHC is a staining technique that uses antibodies to detect the presence of a specific antigen. This study evaluated the expression of pFAK, ROCK1, ROCK2, and pMLC(MYL9) proteins using IHC [28, 46]. Firstly, 4 μm sectioned tissues were cut from paraffin-embedded lung tissue and placed on charged slides. Afterward, the tissues are deparaffinized in xylene, rehydrated in absolute alcohol, and undergo antigen retrieval in 1 × 10 mM sodium citrate buffer (Merck, Germany). Then, the tissues were blocked in 3% H_2_O_2_ (Merck, Germany), followed by first incubation with 10% normal goat serum (NGS) and second incubation overnight at 4 °C with rabbit anti-pFAK polyclonal antibody (diluted to 1:100; Catalog No. orb304528; Biorbyt, UK), rabbit anti-ROCK1 polyclonal antibody (diluted to 1:100; Catalog No. HPA007567; Sigma-Aldrich, USA), rabbit anti-ROCK2 polyclonal antibody (diluted to 1:100; Catalog No. HPA007459; Sigma-Aldrich, USA), and rabbit anti-pMLC(MYL9) polyclonal antibody (diluted to 1:100; Catalog No. PA5-17727; Invitrogen, USA). The negative tissues were incubated with 10% NGS only, without primary antibodies. The tissues were incubated with 3, 30-Diaminobenzidine (DAB) (Dako, Glostrup, Denmark) for peroxidase detection and counterstained with hematoxylin. Finally, the tissues were dehydrated with alcohol and xylene before being mounted with DPX. The percentage of DAB pixel intensity (brown staining) in the cytoplasm or in both cytoplasm/nucleus, which indicated positive immunoreaction, was analysed semi-quantitatively using FIJI-ImageJ 1.52p software (Java 8 version 64-bit) (NIH, Bethesda, MD, USA).

### Western blot

Proteomic ROCK signaling pathway activation was assessed by using Western blot. Firstly, lung tissues were homogenized in RIPA lysis buffer, supplemented with protease inhibitor (Merck, Germany) and phosphatase inhibitor cocktail (Bio basic, Canada). The protein concentration was determined via the Bicinchoninic acid assay (Merck, Germany). 20 µg of protein lysate was separated with 12% SDS-PAGE and transferred to a PVDF membrane. Then, the membrane was blocked with 5% bovine serume albumin and incubated overnight at 4 °C with rabbit anti-pFAK polyclonal antibody (diluted to 1:1000; Catalog No. orb304528; Biorbyt, UK), mouse anti-RhoABC monoclonal antibody (diluted to 1:1000; Catalog No. MA1-011; Invitrogen, USA), rabbit anti-ROCK1 polyclonal antibody (diluted to 1:1000; Catalog No. HPA007567; Sigma-Aldrich, USA), rabbit anti-ROCK2 polyclonal antibody (diluted to 1:1000; Catalog No. HPA007459; Sigma-Aldrich, USA), and rabbit anti-pMLC(MYL9) polyclonal antibody (diluted to 1:1000; Catalog No. PA5-17727; Invitrogen, USA). Then, the membrane was incubated with Horseradish peroxidase-conjugated secondary antibody for 1 h. Beta-actin was used as a housekeeping protein. The signal volume (protein band) was quantitatively measured using Fusion FX-Vilber Lourmet software.

### Microarray data acquisition and differentially expressed genes identification

To further evaluate the potential involvement of pFAK, RhoABC, ROCK1, ROCK2, and pMLC(MYL9) during carcinogenesis of LUSC in human, a bottom-up approach was conducted by investigating the differential expression of the respective. The gene expression profiles of LUSC were retrieved from Gene Expression Omnibus (GEO; https://www.ncbi.nlm.nih.gov/geo/) [51] using the keywords search as follows: “lung”, “cancer”, and “squamous”. The search results were filtered by considering the datasets from the microarray study and the tissue samples of *Homo sapiens*. The datasets were limited to squamous lung cancer samples. Two gene expression profile data of GSE30219 and GSE31446 were obtained in LUSC tissues for the subsequent differentially expressed genes (DEGs) analysis. The microarray data of GSE30219 were obtained using the GPL570 platform ([HG-U133_Plus_2] Affymetrix Human Genome U133 Plus 2.0 Array), derived from 14 non-tumoral lung samples and 61 LUSC samples [52]. GSE31446, on the other hand, was performed in the GPL9244 platform (DKFZ SC123 / Operon Human 38k V4.0b – r1), included 64 samples derived from the inner tumor (IT; *n* = 16), tumor invasion front (TI; *n* = 18), adjacent lung front (LF; *n* = 15) and normal lung (NL; *n* = 15) [53]. The samples from GSE31446 were categorized into LUSC (IT and TI) and non-tumoral lung samples (NL and LF) before being analysed.

In this study, a DEG analysis was conducted on the GSE30219 and GSE31446 datasets using the GEO2R tool (https://www.ncbi.nlm.nih.gov/geo/geo2r/) [54]. GEO2R adopts the limma R package to analyse the expression profiling by array data [55]. The DEGs between the LUSC and non-LUSC (healthy control) groups were identified separately for each dataset, given the different array platforms. Parameters for the DEGs identification were as follows: |Log_2_FC| > 1 and *p*-adjust value < 0.05. DEGs were considered significantly upregulated if Log_2_FC *≥* 1, and downregulated if Log_2_FC *≤* -1. The Benjamini & Hochberg false discovery rate method was applied to correct false positive results. The expressions of pFAK(PTK2), ROCK1, ROCK2, and pMLC(MYL9) were subsequently observed to infer their roles in human LUSC.

### Network-based prediction of pFAK(PTK2), RhoABC, ROCK1, ROCK2, and pMLC(MYL9) protein interactions

The protein-protein interaction (PPI) data of targeted proteins were retrieved from STRING (https://string-db.org) [56]. STRING is a database that stores integrated PPI data derived from physical interactions and functional associations (e.g., co-expression, gene neighborhood, gene fusion, and gene co-occurrence). pFAK(PTK2), RhoA, RhoB, RhoC, ROCK1, ROCK2, and pMLC(MYL9) were submitted as query proteins to the STRING database. The interaction data for STRING was obtained by applying a confidence score of 0.4 and a false discovery rate (FDR) stringency of 5%. The networks were visualized using Cytoscape v3.9.1 software [57].

### Correlation analysis of pFAK(PTK2), RhoABC, ROCK1, ROCK2, and pMLC(MYL9) in human LUSC

Correlation analysis was performed to verify the relationship between pFAK(PTK2), RhoABC, ROCK1, ROCK2, and pMLC(MYL9) gene expression levels in human LUSC. The analysis was performed using Gene Expression Profiling Interactive Analysis 2 (GEPIA2; http://gepia2.cancer-pku.cn/#index) [58]. GEPIA2 provides functionality for analysing and comparing expression data across a large collection of TCGA and GTEx samples. To compute the correlation between pFAK(PTK2), RhoABC, ROCK1, ROCK2, and pMLC(MYL9) interactions in LUSC tissues, we performed the Pearson Correlation Coefficient (PCC) method, and a *p*-value < 0.001 was considered as a significant correlation among these genes. The LUSC tumor and LUSC normal were selected as reference expression datasets in GEPIA2.

### Statistical analysis

All data were expressed as mean ± standard error of the mean (SEM) of at least three biological replicates for the IHC and Western blot assays. All statistical analyses for IHC and Western blot were conducted and analysed using GraphPad Prism version 8.3.0. Students’ t-test was applied to investigate the significant differences between groups. Statistically significant differences were considered when *p*-value < 0.05.

## Results

### pFAK, ROCK1, ROCK2, and pMLC(MYL9) protein expression were increased during LUSC carcinogenesis as determined by IHC staining

**Fig. 1:**
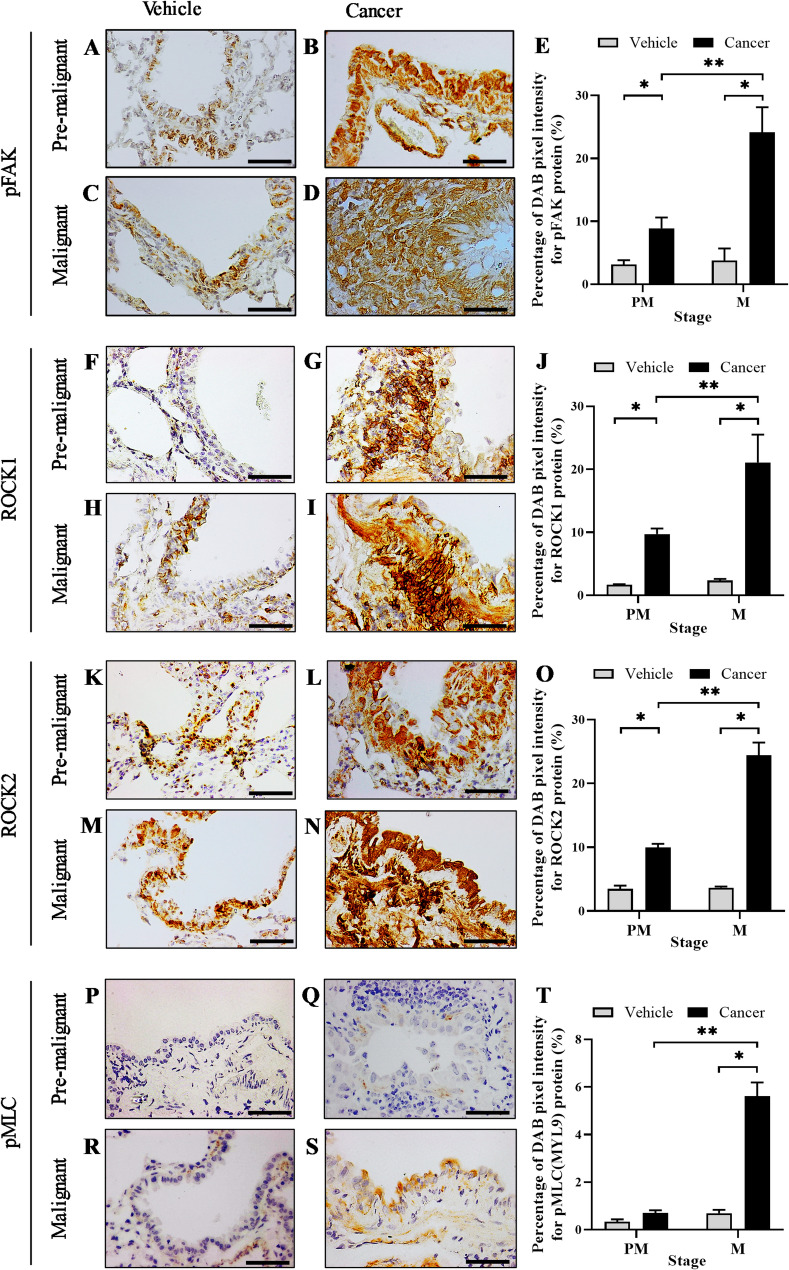
IHC Staining for pFAK, ROCK1, ROCK2 and pMLC(MYL9) at the bronchus/bronchial from the Vehicle (**A**, **C**, **F**, H, **K**, **M**, **P**, and **R**) and Cancer (**B**, **D**, **G**, **I**, **L**, **N**, **Q**, and **S**) groups obtained from the pre-malignant and malignant stage at 40X magnification. Brown staining represents positive pFAK, ROCK1, ROCK2 and pMLC(MYL9) protein immunoreaction. **E**, **J**, **O**, and **T** show the bar chart for the percentage of DAB pixel intensity for pFAK, ROCK1, ROCK2 and pMLC(MYL9) protein (%), respectively. Scale bars=50 µm. *Significant difference (*p*-value<0.05) between the Vehicle and Cancer groups in the same stage. **Significant difference (*p*-value<0.05) between the Cancer groups from two different stages of carcinogenesis

In this study, IHC staining for pFAK, ROCK1, ROCK2, and pMLC(MYL9) was performed to determine ROCK signaling pathway activation and protein localization in cells. From this assay, pFAK from the PM and M Cancer groups (Fig. 1-B and D) was observed to have a more intense positive brown DAB staining as compared to their respective Vehicle groups in both nucleus and cytoplasm; (Fig. 1-A and C). Similarly, ROCK1 (Fig. 1-G and I) and ROCK2 (Fig. 1-L and N) from the PM and M Cancer groups were also observed to show more intense positive brown DAB staining as compared to their respective Vehicle groups; (Fig. 1-F and H) for ROCK1 and (Fig. 1-K and M) for ROCK2. ROCK1 was observed to be localized in the plasma membrane, cytoplasm, and nucleus (particularly in the M Cancer group). Meanwhile, ROCK2 was observed to be localized in the nucleus and cytoplasm. Additionally, for pMLC(MYL9) protein, an intense cytoplasmic positive brown DAB staining is only observed in the M Cancer group (Fig. 1-S) as compared to the M Vehicle group (Fig. 1-R) and PM groups; both Cancer and Vehicle groups (Fig. 1-P and Q).

Qualitatively, pFAK protein expression was significantly higher (*p*-value < 0.05) in the PM Cancer group as compared to the PM vehicle group, with percentages of DAB pixel intensity of 8.88 ± 1.74% and 3.14 ± 0.69%, respectively (Fig. 1-E). Similarly, the M Cancer group has a significantly higher (*p*-value < 0.05) pFAK protein expression as compared to the M Vehicle group, with percentages of DAB pixel intensity of 24.15 ± 3.98% and 3.76 ± 1.93%, respectively (Fig. 1-E). The PM Cancer group has a significantly higher (*p*-value < 0.05) ROCK1 protein expression as compared to the PM Vehicle group as indicated by percentages of DAB pixel intensity of 9.73 ± 0.88 and 1.69 ± 0.09%, respectively (Fig. 1-J). Similarly, the M Cancer group also has a significantly higher (*p*-value < 0.05) ROCK1 protein expression as compared to the M Vehicle group as indicated by percentages of DAB pixel intensity of 21.09 ± 4.43% and 2.35 ± 0.27%, respectively. The same trend was also observed for ROCK2 protein (Fig. 1-O). The percentage of DAB pixel intensity for ROCK2 in the PM Cancer group was found to be significantly higher (*p*-value < 0.05), which is 9.98 ± 0.56% as compared to the PM Vehicle group; 3.50 ± 0.49%. While the percentage of DAB pixel intensity for ROCK2 in the M Cancer group was found to be significantly higher (*p*-value < 0.05), which is 24.45 ± 1.97% as compared to the M Vehicle group; 3.67 ± 0.16%. For pMLC(MYL9) protein (Fig. 1-T), the percentage of DAB pixel intensity was only found to be significantly higher (*p*-value < 0.05) in the M Cancer group, which is 5.61 ± 0.58% as compared to the M Vehicle group; 0.7 ± 0.1%. Collectively, IHC assay showed increasing trend of ROCK signalling pathway expression from the pre-malignant to malignant stages as shown by significantly higher (*p*-value < 0.05) pFAK, ROCK1, ROCK2, and pMLC(MYL9) protein expression in the M Cancer groups as compared to the PM Cancer groups. 

### pFAK, RhoABC, ROCK1, ROCK2 and pMLC(MYL9) protein expression were increased during LUSC carcinogenesis as determined by Western blot

**Fig. 2:**
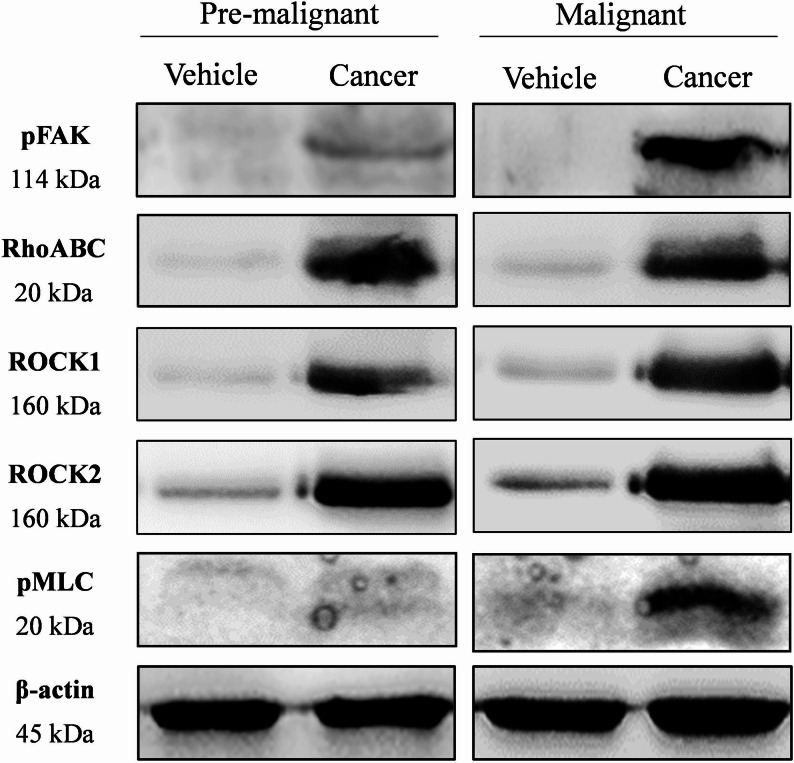
Protein band from each group shows the intensity of the protein expression of pFAK, RhoABC, ROCK1, ROCK2, and pMLC(MYL9), analysed from the homogenate of pre-malignant and malignant LUSC tissue. The Pre-malignant and Malignant blots for each protein were grouped and cropped from different PVDF membranes

For this assay, the protein expression of pFAK, RhoABC, ROCK1, ROCK2, and pMLC(MYL9) were evaluated from the PM and M LUSC tissues homogenate. Based on Fig. 2, a thicker band of pFAK, RhoABC, ROCK1, and ROCK2 indicating higher protein expression was observed in most PM Cancer groups as compared to the PM Vehicle groups. Similarly, the bands were also thicker in all M Cancer groups as compared to the M Vehicle groups.

**Fig. 3:**
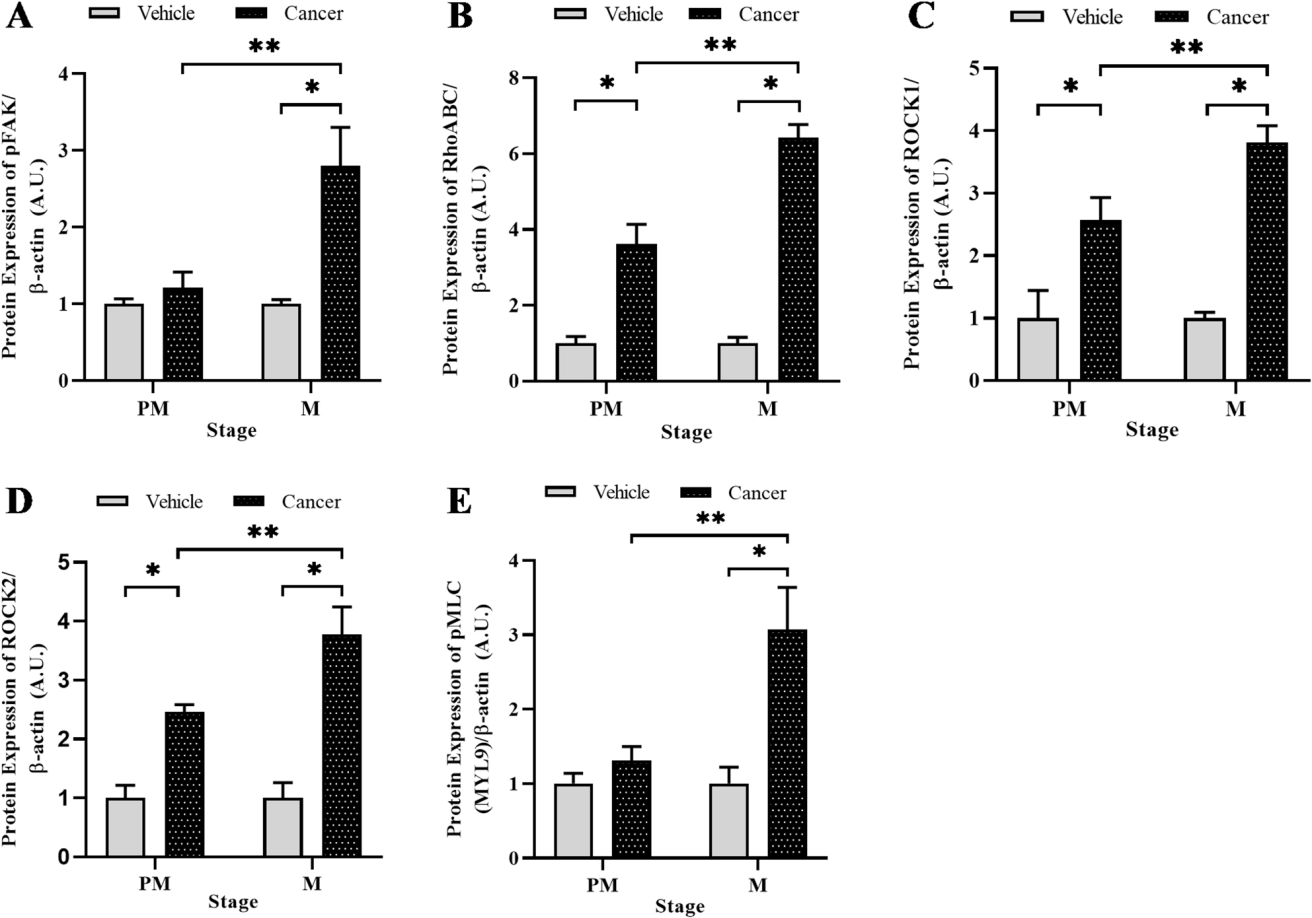
The quantitative bar chart of protein expression of (**A**) pFAK, (**B**) RhoABC, (**C**) ROCK1, (**D**) ROCK2, and (**E**) pMLC(MYL9) in arbitrary unit (A.U.) from protein band. *Significant difference (*p*-value < 0.05) between the Vehicle and Cancer groups in the same stage. **Significant difference (*p*-value < 0.05) between Cancer groups from two different stages of carcinogenesis

pFAK protein expression was elevated in the Cancer group as compared with the Vehicle group at both stages of carcinogenesis. Nevertheless, the expression of pFAK in the Cancer group was only found to be significantly higher (*p*-value < 0.05) in the M stage, which is 2.80 ± 0.50 A.U. as compared to the M Vehicle group; 1.00 ± 0.05 A.U. For RhoABC (Fig. 3-B), the protein expression was found to be significantly higher (*p*-value < 0.05) in the PM Cancer group, which is 3.63 ± 0.53 A.U. as compared to the PM Vehicle group; 1.00 ± 0.17 A.U. Similarly, the protein expression of RhoABC was found to be significantly higher (*p*-value < 0.05) in the M Cancer group, which is 6.43 ± 0.34 A.U. as compared to the M Vehicle group; 1.00 ± 0.16 A.U (Fig. 3-C).

The same trend was also observed for ROCK1 (Fig. 3-C) and ROCK2 protein expression (Fig. 3-D). The protein expression of ROCK1 in the PM Cancer group was found to be significantly higher (*p*-value < 0.05), which is 2.57 ± 0.36 A.U. as compared to the PM Vehicle group; 1.00 ± 0.44 A.U. While, the protein expression of ROCK1 in the M Cancer group was found to be significantly higher (*p*-value < 0.05), which is 3.81 ± 0.27 A.U. as compared to the M Vehicle group; 1.00 ± 0.93 A.U. For ROCK2, the PM Cancer group and M Cancer group were also found to have a significantly higher (*p*-value < 0.05) protein expression, which is 2.46 ± 0.12 A.U. and 3.78 ± 0.47 A.U., respectively as compared to the Vehicle groups, which is 1.00 ± 0.22 A.U for PM Vehicle and 1.00 ± 0.26 A.U. for the M Vehicle group.

Whereas, for pMLC(MYL9) (Fig. 3-E), the protein expression was only found to be significantly higher (*p*-value < 0.05) in the M Cancer group, which is 3.778 ± 0.467 A.U. as compared to the M Vehicle group; 1.00 ± 0.26 A.U. Collectively, Western blot result showed increasing trend of ROCK signaling pathway expression from the pre-malignant to malignant stages as shown by significantly higher (*p*-value < 0.05) pFAK, RhoABC, ROCK1, ROCK2, and pMLC(MYL9) protein expression in the M Cancer groups as compared to the PM Cancer groups. 

### Identification of DEGs and establishment of pFAK(PTK2), RhoABC, ROCK1, ROCK2, and pMLC(MYL9) PPI network

**Fig. 4:**
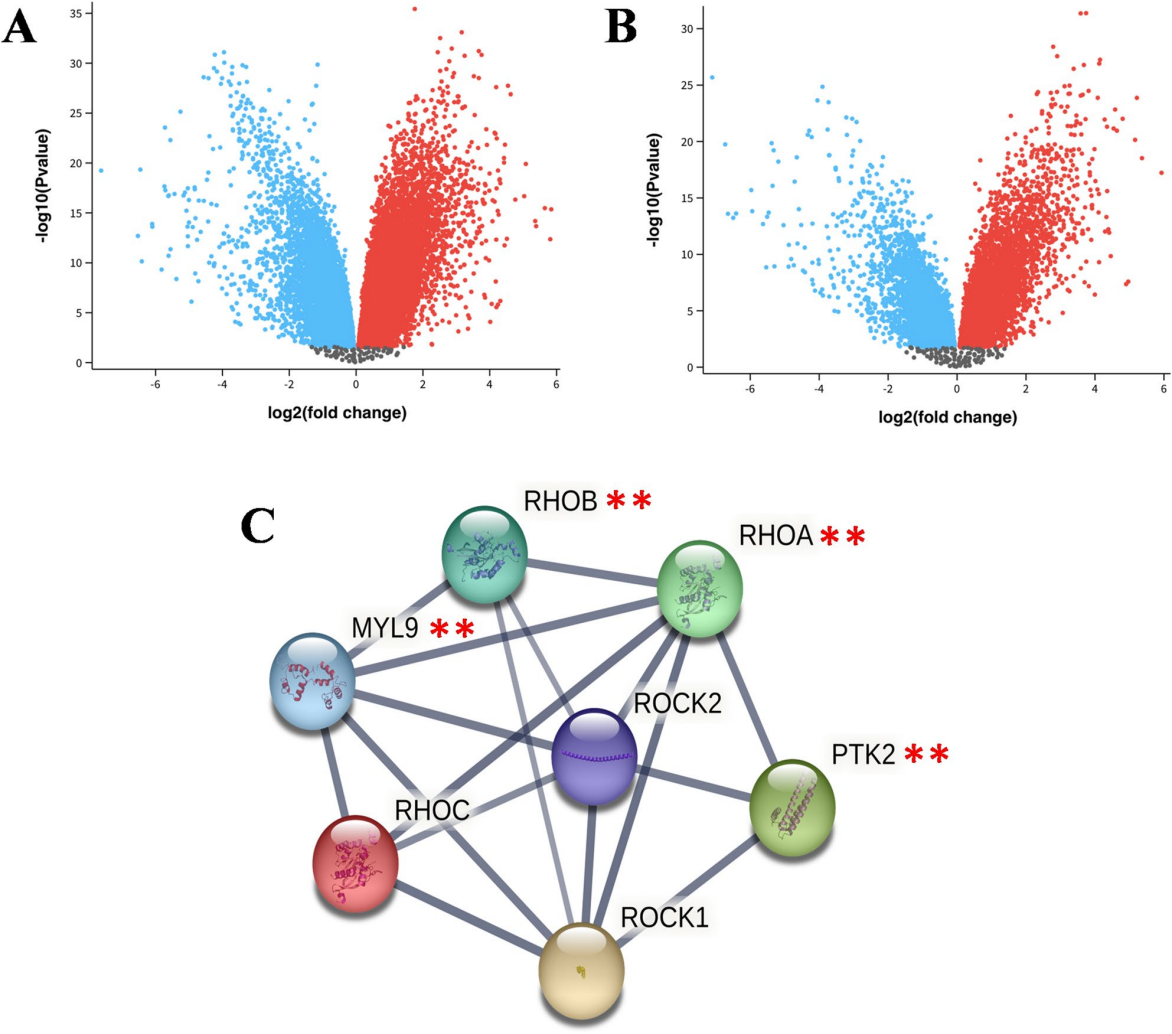
Identification of DEGs in two expression profiling datasets: (**A**) GSE30219 and (**B**) GSE31446. **C** The network represents interactions between pFAK(PTK2), RhoABC, ROCK1, ROCK2, and pMLC(MYL9). ** symbol indicates the significant DEGs found in either one of the datasets

Based on the GEO2R analysis, 5205 and 2614 DEGs were discovered from the GSE30219 and GSE31446 datasets, respectively. In the study, GSE30219 exhibited 3180 upregulated and 2025 downregulated DEGs, whereas GSE31446 showed 1630 upregulated and 984 downregulated DEGs. Figure 4-A and -B represent the volcano plot of the significant DEGs with |Log_2_FC| > 1 and *p*-adjust value < 0.05. Among them, three DEGs, namely RhoA, RhoB, and pMLC(MYL9), were found to be significantly upregulated, and pFAK(PTK2) was downregulated in human LUSC (Table 1). The network involving all seven DEGs was constructed, as shown in Fig. 4-C. The network consisted of seven nodes and 17 edges. RhoA, ROCK1, and ROCK2 had the highest interaction among them, *n* = 6, followed by pMLC(MYL9) (*n* = 5), RhoB and RhoC (*n* = 4), and pFAK(PTK2) (*n* = 3). The confidence score (CS) *≥* 0.9 was used to observe the reliability of PPI, and three interaction pairs were discovered to have medium interaction confidence, such as RhoC–ROCK2 (CS = 0.805), RhoB–ROCK2 (CS = 0.69), and RhoB–ROCK1 (CS = 0.69). Detailed information on the 17 PPI pairs is shown in Table SI.

**Table 1:** List of DEGs identified in human LUSC datasets (GSE30219 and GSE31446)

gene name	Gene identity	GSE30219		GSE31446	
		*P*-adj value	Log_2_FC	*P*-adj value	Log_2_FC
pFAK(PTK2) **	5747	**2.17 × 10** ^**− 10**^	**-1.59**	7.42 × 10^− 06^	7.48 × 10^− 01^
RhoA **	387	4.34 × 10^− 09^	4.87 × 10^− 01^	**1.18 × 10** ^**− 07**^	**1.29**
RhoB **	388	**2.47 × 10** ^**− 11**^	**1.63**	6.31 × 10^− 06^	5.47 × 10^− 01^
RhoC	389	1.23 × 10^− 01^	1.90 × 10^− 01^	7.74 × 10^− 01^	4.61 × 10^− 02^
ROCK1	6093	7.37 × 10^− 04^	4.02 × 10^− 01^	9.69 × 10^− 01^	2.40 × 10^− 03^
ROCK2	9475	1.01 × 10^− 04^	5.17 × 10^− 01^	1.07 × 10^− 05^	9.69 × 10^− 01^
pMLC(MYL9) **	10,398	**2.90 × 10** ^**− 10**^	**1.82**	**1.28 × 10** ^**− 05**^	**1.97**

DEGs with ** symbol indicate significant DEGs in at least one dataset, where |Log2FC| > 1 and *p*-adjust value < 0.05

DEGs with ** symbol indicate significant DEGs in at least one dataset, where |Log2FC| > 1 and p-adjust value < 0.05.

### Correlation analysis of pFAK(PTK2), RhoABC, ROCK1, ROCK2, and pMLC(MYL9) in TCGA LUSC

**Fig. 5:**
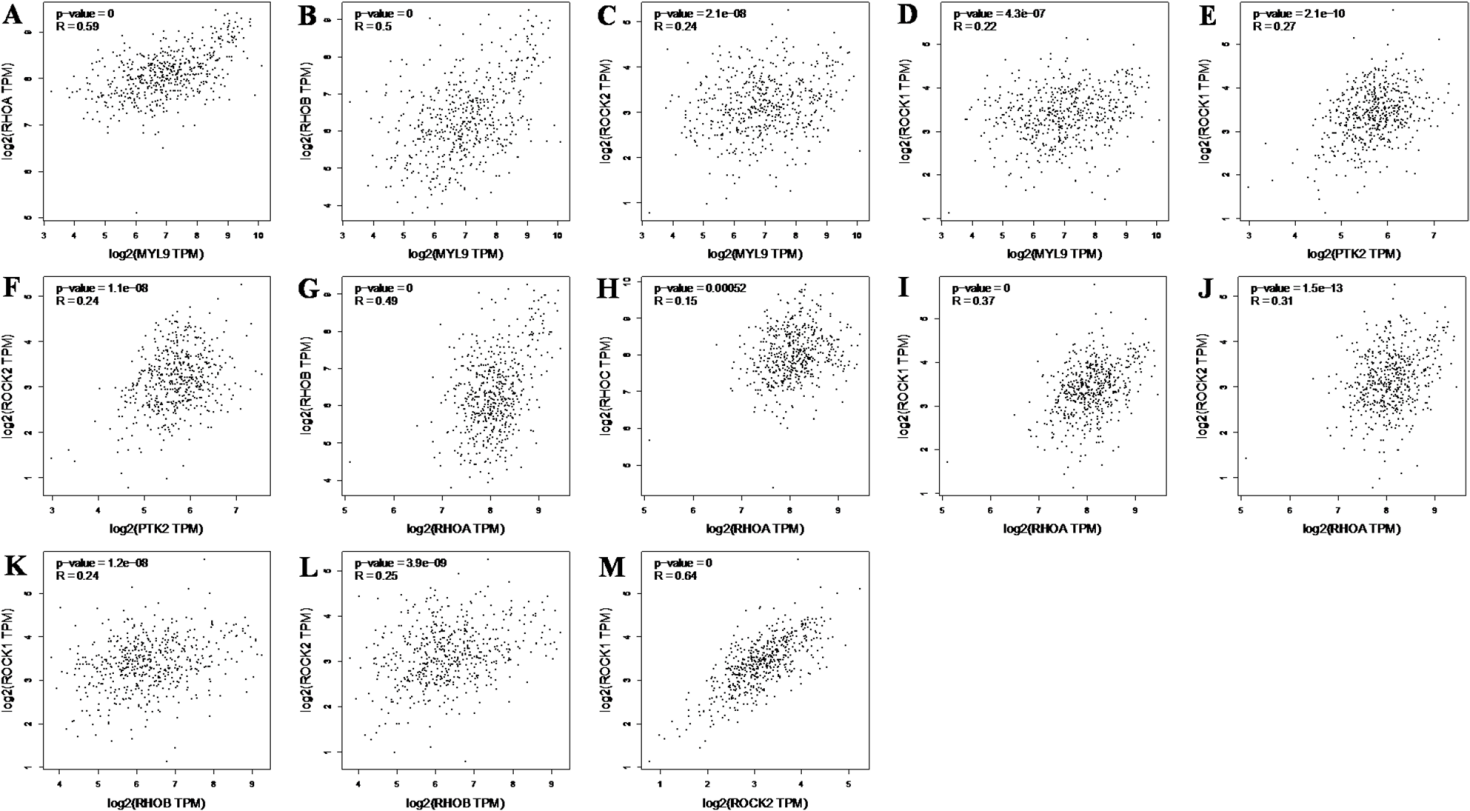
Significant correlation of pFAK(PTK2), RhoABC, ROCK1, ROCK2, and pMLC(MYL9) interaction pairs using GEPIA2. **A** pMLC(MYL9)–RhoA; **B** pMLC(MYL9)–RhoB; **C** pMLC(MYL9)–ROCK2; **D** pMLC(MYL9)–ROCK1; **E** pFAK(PTK2)–ROCK1; **F** pFAK(PTK2)–ROCK2; **G** RhoA–RhoB; **H** RhoA–RhoC; **I** RhoA–ROCK1; **J** RhoA–ROCK2; **K** RhoB–ROCK1; **L** RhoB–ROCK2; and (**M**) ROCK1–ROCK2. The insignificant correlation between interaction pairs of pMLC(MYL9)–RhoC, pFAK(PTK2)–RhoA, RhoC–ROCK1, and RhoC–ROCK2 are not shown

To verify the relationship between pFAK(PTK2), RhoABC, ROCK1, ROCK2, and pMLC(MYL9) expression levels, we conducted a correlation analysis on the 17 interaction pairs of seven genes-encoded proteins. The results revealed that 13 interacting genes-encoded proteins pairs were positively correlated in LUSC (Fig. 5). We observed a positive correlation between pFAK(PTK2) with ROCK1 (*p*-value = 2.1 × 10-10, *R* = 0.27) and ROCK2 (*p*-value = 1.1 × 10-08, *R* = 0.24). RhoA was positively correlated with RhoB (*p*-value = 0, *R* = 0.49), RhoC (*p*-value = 0.00052, *R* = 0.15), ROCK1 (*p*-value = 0, *R* = 0.37), and ROCK2 (*p*-value = 1.5 × 10-13, *R* = 0.31), whereas RhoB positively correlated with ROCK1 (*p*-value = 1.2 × 10-08, *R* = 0.24) and ROCK2 (*p*-value = 3.9 × 10-09, *R* = 0.25). pMLC(MYL9) expression, on the other hand, was positively correlated with RhoA (*p*-value = 0, *R* = 0.59), RhoB (*p*-value = 0, *R* = 0.5), ROCK2 (*p*-value = 2.1 × 10-08, *R* = 0.24), and ROCK1 (*p*-value = 4.3 × 10-07, *R* = 0.22). A positive correlation was also observed for ROCK1–ROCK2 (*p*-value = 0, *R* = 0.64). Four interaction pairs, such as pMLC(MYL9)–RhoC, pFAK(PTK2)–RhoA, RhoC–ROCK1, and RhoC–ROCK2, were not correlated in LUSC based on *p*-value > 0.001.

## Discussion

Rho-associated kinase (ROCK) is critical for regulating the dynamics of the actin skeleton, which controls various cancer cell phenotypes, especially cell motility and migration [59]. Thus, in-depth understanding of ROCK-associated genes and their encoded proteins expression, interactions, and correlation is essential, especially in deadly cancers like LUSC. Since the introduction of NTCU-induced LUSC in mouse model for pre-clinical studies, research on LUSC has greatly increased, thanks to its high reproducibility and reliability [44, 46, 50]. Therefore, the use of NTCU-induced LUSC in vivo for evaluating specific pathway alterations, such as ROCK gene and protein expression can provide a better understanding of the disease.

In this study, we analysed the expression of ROCK-associated proteins to predict ROCK signaling pathway activation, as most phenotypic effects were regulated by protein. Hence, we determined the expression of proteins associated with ROCK including ROCK1/ROCK2, the upstream effectors; pFAK and RhoABC, and the downstream substrate; pMLC(MYL9). Briefly, our NTCU-induced LUSC mice demonstrated a higher pFAK, ROCK1, ROCK2, and pMLC(MYL9) expression in both the pre-malignant and malignant LUSC via IHC staining. The expression of these proteins was also observed to increase during carcinogenesis, as indicated by a significant increase in percentage of DAB pixel intensity (%) for pFAK, ROCK1, ROCK2, and pMLC from pre-malignant to malignant groups. This evidence suggested activation of the ROCK signaling pathway and its profound roles in LUSC progression. The localization of protein expression was consistent with previous studies that have identified the presence of pFAK and pMLC(MYL9) proteins in the cytoplasm [60, 61], ROCK1 protein in the cytoplasm, nucleus, or around plasma membrane [62], and ROCK2 protein in the nucleus or cytoplasm [63], through IHC staining. The localized protein expression of ROCK1 around the plasma membrane is attributable to its primary function in cell polarity [64]. Meanwhile, to the best of our knowledge, reports regarding nuclear localization of ROCK1 in the cancer cells remain limited in the literature. However, translocation from the cytoplasm to the nucleus has been observed in a study as ROCK1 hinders the egress of human cytomegalovirus (HCMV) from the nucleus in HCMV-infected cells [65]. Notably, the expression of ROCK2 in the nucleus is due to its interaction with pSTAT and p300, the primary regulator of promoter activity and chromatin acetylation located in the nucleus [66].

The evaluation of ROCK-associated protein expression was further confirmed by Western blot with the addition of RhoABC protein. Generally, Western blot results showed a similar pFAK, ROCK1, ROCK2, and pMLC(MYL9) proteins expression pattern obtained from IHC staining, especially at the malignant stage. As observed in this study, high pFAK expression has also been reported in the tissue of patients with small cell lung cancer [67, 68], adenocarcinoma [68, 69], and LUSC subtype [70]. Notably, our finding of higher pFAK expression, especially in the malignant stage LUSC, agreed with previous studies that reported a positive correlation between pFAK with tumor size and metastasis involving lymph nodes [69, 70]. pFAK protein expression was an important ROCK effector to be analysed since it is one of the primary ‘biosensor’ that translate the extracellular forces (E.g., increased tissue stiffness) experienced by cancer cells into intracellular oncogenic signaling pathways, primarily ROCK [71]. Cancer was accompanied by increased tissue rigidity due to over-synthesis of collagen, and increased proliferation of cancer and stromal cells [72, 73], which was suggested to increase pFAK-ROCK expression in cancer [12, 19]. The pFAK protein then activates RhoABC, a combination of small GTPase proteins RhoA, RhoB, and RhoC. RhoA/B/C shares 88% amino acid identity, which is capable of binding to Rho-binding region of the ROCK domain. They also share similar amino acid sequences crucial for catalytic activity, such as Gly14, Thr19, Phe30, and Gln63 [74]. While RhoA is widely recognized as the primary activator of ROCK1/2, research has shown that RhoB and RhoC are also capable of activating ROCK1/2 to an equivalent extent (17). This rationale underscores our decision to choose RhoABC for Western blot, justifying RhoABC evaluation for the protein expression analysis in our study. According to Wang et al. [75] and Xu et al. [76], RhoA expression was found to be highly expressed in NSCLC cancer cells and lung cancer tissues. Furthermore, the finding of higher RhoABC expression in malignant stage LUSC tissue is in accordance with the study done by Zhang et al. [77] and Du et al. [32], who found higher RhoA expression in late-stage (Stage III and IV) as compared to early-stage (Stage I and II) NSCLC cancer.

ROCK is a critical protein in ROCK signaling pathway, activated by RhoABC [78]. ROCK1 or ROCK2 can activate several downstream targets, including MYPT, MLC(MYL9), and LIMK1/2 [79, 80]. In this study, both ROCK1 and ROCK2 expressions were evaluated as previous studies showed inconsistent expression of both ROCK isoforms in different types of cancer. For example, ROCK1 was reported to be highly expressed in osteosarcoma, breast, brain, and ovarian cancers [19, 81–83]. Meanwhile, ROCK2 was reported to be highly expressed in skin cancer, neuroblastoma, and liver [28, 84, 85]. For lung cancer, both ROCK isoforms have been identified to be highly expressed. Specifically, ROCK1 was reported to be highly expressed in NSCLC cells and tissues [29, 86, 87]. ROCK1 overexpression is reported to have a positive and significant correlation with tumor size, advanced tumor, and low survival rate, as analysed in NSCLC tissues [29]. ROCK1 protein expression was also found to be significantly higher in the late-stage than in an early-stage NSCLC [29, 32], which is in line with our finding that observed high ROCK1 protein expression, especially in the malignant stage. The role of ROCK2 in promoting cancer growth is also undeniably important, given that ROCK2 is highly expressed in our LUSC tissue, which was found to increase during carcinogenesis. Although the study of its expression in lung cancer cells or tissues remains poorly characterized, ROCK2 has been reported to support cell cycle progression, development, and ECM proteins deposition in lung cancer [88, 89]. According to Ye et al. [36], ROCK2 can stimulate the epithelial-mesenchymal transition process in cisplatin-resistant NSCLC by reducing the expression of miR-101. Furthermore, ROCK2 inhibition has been reported to have the same debilitating effect on lung cancer cell growth as ROCK1 inhibition [31]. In conclusion, both ROCK isoforms are proposed to have an equally important and independent roles in promoting and LUSC growth.

pMLC(MYL9) is one of the last or main downstream substrate proteins of ROCK signaling pathway, evaluated in this study. The pMLC(MYL9) protein expression was evaluated due to its ability to directly activate myosin ATPase. This activation supports the interaction between myosin II and F-actin, which affects actomyosin contraction and eventually, cell behavior [26, 90]. Meanwhile, MYPT and LIMK1/2 proteins only function as support proteins that regulate MLC(MYL9) phosphorylation and the interaction between myosin II and F-actin. Active MYPT and LIMK1/2 proteins act as inhibitors for MLC(MYL9) dephosphorylation and mediators for actin polymerization, respectively [14]. As observed by IHC staining and Western blot, pMLC(MYL9) expression is significantly elevated only at the malignant stage. This finding could be attributed to pMLC(MYL9)’s prominent role in the malignant phenotype of cancer cells, such as migration, which is an essential step for cancer cell invasion and metastasis in late-stage cancer [91, 92]. Our finding is in line with a previous study conducted by Wang et al. [93] who found a positive and significant correlation between pMLC(MYL9) expression and PFN-1; a protein highly expressed in late-stage NSCLC tissues. In addition, high pMLC(MYL9) expression was also found to have a significant relationship with late-stage NSCLC cancer involving lymph node metastasis [94]. Overall, ROCK-associated protein expression, as assessed by IHC and Western blot showed an increasing trend from pre-malignant to malignant stages. This increase in protein expression throughout carcinogenesis suggests a crucial role of ROCK-associated proteins for the development of LUSC, as they sustain multiple hallmarks of cancer [31, 36, 95].

Transcriptomic analysis of DEGs from human LUSC tissue databases verified the dysregulation of ROCK-associated proteins such as pFAK, RhoA, RhoB, and pMLC(MYL9) in LUSC. The upregulation of proteins, particularly pMLC(MYL9), confirmed the involvement of ROCK signaling pathway in LUSC carcinogenesis since pMLC(MYL9) is the final protein that executes the effect on cell behavior [23]. Based on the PPI network analysis result, RhoA, ROCK1, and ROCK2 show the highest number of interactions; *n* = 6, with the other ROCK-associated proteins. Additionally, the frequencies of RhoA, ROCK1, and ROCK2 were among the highest to positively correlate with ROCK-associated proteins. These findings may reflect the profound role of ROCK1/ROCK2 as the core proteins in ROCK signaling pathway. Indeed, most previous studies on Rho-ROCK in cancer have focused solely on RhoA expression [19, 76, 96–98], making it one of the commonly studied Rho GTPase families. For instance, RhoA/ROCK has been reported to play a crucial role in promoting NSCLC cell proliferation in vitro [75, 76, 99] and in vivo [88] studies. In addition, RhoA-ROCK can promote vascular structure (angiogenesis), which is necessary for lung cancer migration and metastasis [37]. Briefly, our findings of PPI interactions and correlations may support the essential role of RhoA-ROCK as a worthy therapeutic target for LUSC. Nevertheless, the roles of other Rho families such as RhoB and RhoC are also important, as proven by their moderate interaction with either ROCK1 or ROCK2. We also observed inconsistencies in pFAK, ROCK1, and ROCK2 expression between data from genes-encoded proteins of human LUSC and from proteins in NTCU-induced LUSC tissues in mice. However, the positive correlation between pFAK(PTK2), ROCK1, and ROCK2 with other ROCK-associated proteins in human LUSC, as assessed through PPI was appreciated. The inconsistency between mRNA and protein expression of pFAK(PTK2), ROCK1, and ROCK2 may be due to several factors, such as sample heterogeneity, post-transcriptional regulation [100], translation efficiency [101], and alternative splicing [102].

Briefly, our study is among the earliest to confirm ROCK signaling pathway activation in dual-stage carcinogenesis of NTCU-induced LUSC tissue. ROCK signaling pathway activation was proven by increased pFAK, RhoABC, ROCK1, and ROCK2 protein expression in the Cancer groups, especially at the malignant stage of LUSC. pFAK, RhoA, RhoB, and pMLC(MYL9) may be responsible for promoting and nurturing LUSC growth as proven by their significant DEGs dysregulation in human LUSC tissues. While the PPI interaction and correlation analysis suggested the dominant role of RhoA-ROCK1/ROCK2 in ROCK signaling pathway. For future studies, we recommend administering ROCK-associated protein inhibitors, such as fasudil or siRNA, in mice alongside NTCU induction to confirm the role of ROCK signaling pathway in LUSC progression. In-depth study of ROCK signaling pathway in human LUSC tissues is also needed to validate its roles in the carcinogenesis of LUSC. Moreover, research on the tumor microenvironment is essential, as it strongly influences ROCK activation [12]. Herein, advanced approaches such as integrating machine learning to predict dysregulation in tumor microenvironment and in-depth understanding of its relationship with metabolic reprogramming in LUSC, for instance, may provide a robust framework for optimizing personalized treatment in the future [103, 104].

## Conclusion

In conclusion, this study suggests increased ROCK signaling pathway activation during LUSC carcinogenesis in vivo, as evidenced by increased protein expression of pFAK, RhoABC, ROCK1, ROCK2, and pMLC(MYL9) from the pre-malignant to the malignant stage. Additionally, increased expression of both ROCK isoforms; ROCK1 and ROCK2 observed during carcinogenesis in this study might indicate the vital role of both ROCKs in the initiation, progression, and malignancy of LUSC. This study also suggested an increased expression of several ROCK-associated genes-encoded proteins such as RhoA, RhoB, and pMLC(MYL9) in human LUSC by analysing the transcriptome studies, deposited in GEO. The combination of both in vivo and bioinformatic analyses in this study may provide insights and verification regarding the roles of ROCK in LUSC carcinogenesis, which hold potential as novel therapeutic targets in preventing and treating LUSC in the future.

## Supplementary Information

Supplementary Material 1.

## Data Availability

The data supporting the findings of this study are available within the article and its supplementary file.
