# Supplementary material for: Rho-associated kinase (ROCK)-associated proteins and genes-encoded proteins upregulated in lung squamous cell carcinoma (LUSC)
Source: J Egypt Natl Canc Inst. 2026 Apr 20;38:12. doi: 10.1186/s43046-026-00351-0 (PMC13313306; doi:10.1186/s43046-026-00351-0)
Supplement: Supplementary file 1 — Supplementary Material 1. [file 43046_2026_351_MOESM1_ESM.docx]

**Manuscript Title: Rho-Associated Kinase (ROCK)-Associated Proteins and Genes-Encoded Proteins Upregulated in Lung Squamous Cell Carcinoma (LUSC)**

**Supplementary File 1: PVDF membrane of Western Blot**

N = Normal tissue

T = Tumor tissue

Red box = Band choosen for display in main text article

1. Beta-actin and pre-malignant pFAK

**(Beta-actin)**

**T2**

**T3**

**T1**

**N3**

**N2**

**N1**


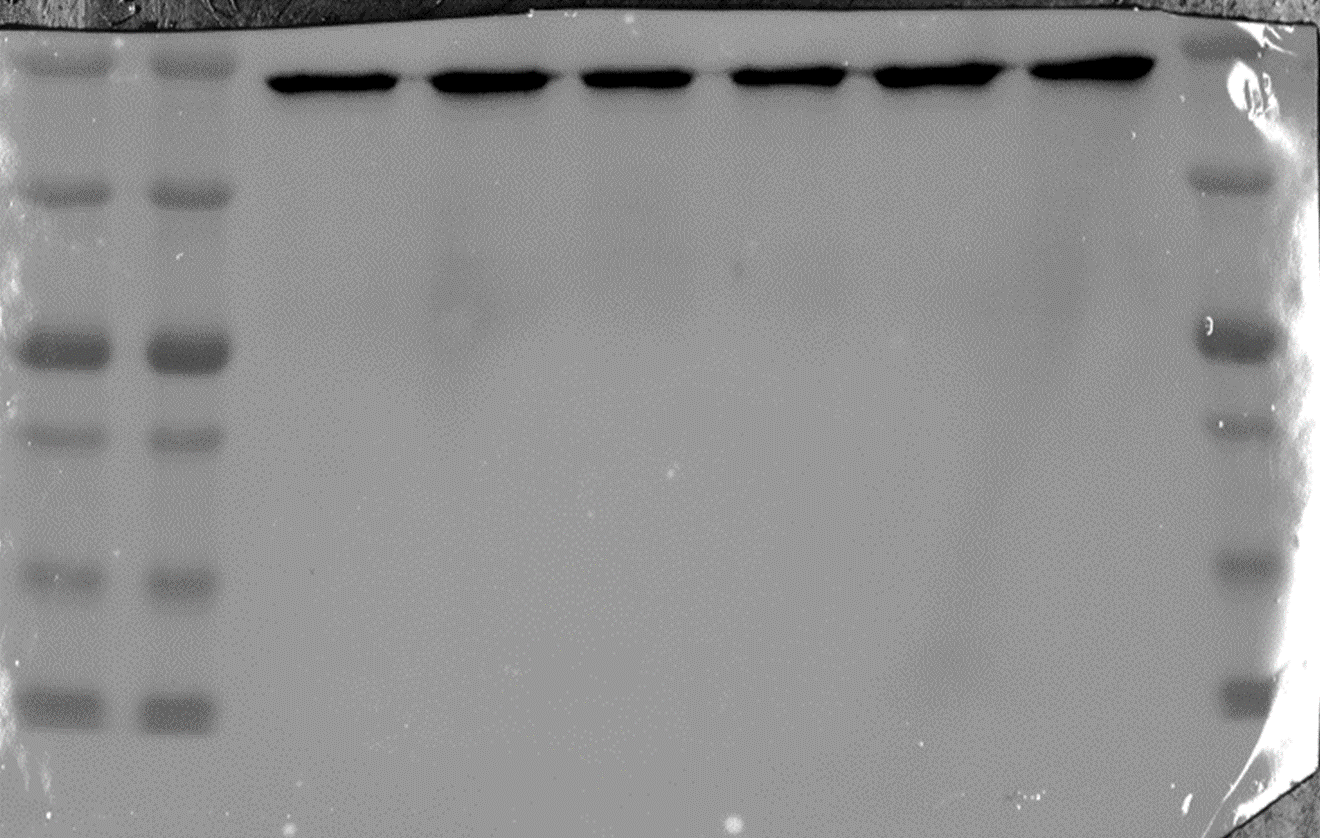


45 kDa

Original picture for **pre-malignant Beta-actin** shown above





Original picture for **(Pre-malignant pFAK)**

**N1**

**N2**

**N3**

**T1**

**T2**

**T3**


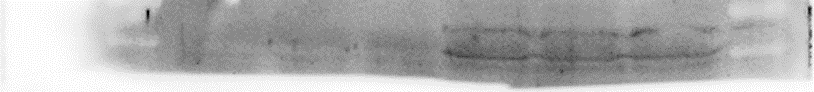


114 kDa

Original picture for **pre-malignant pFAK** shown above





1. Beta-actin and malignant pFAK

**(Beta-actin)**

**T3**

**T2**

**T1**

**N3**

**N2**

**N1**


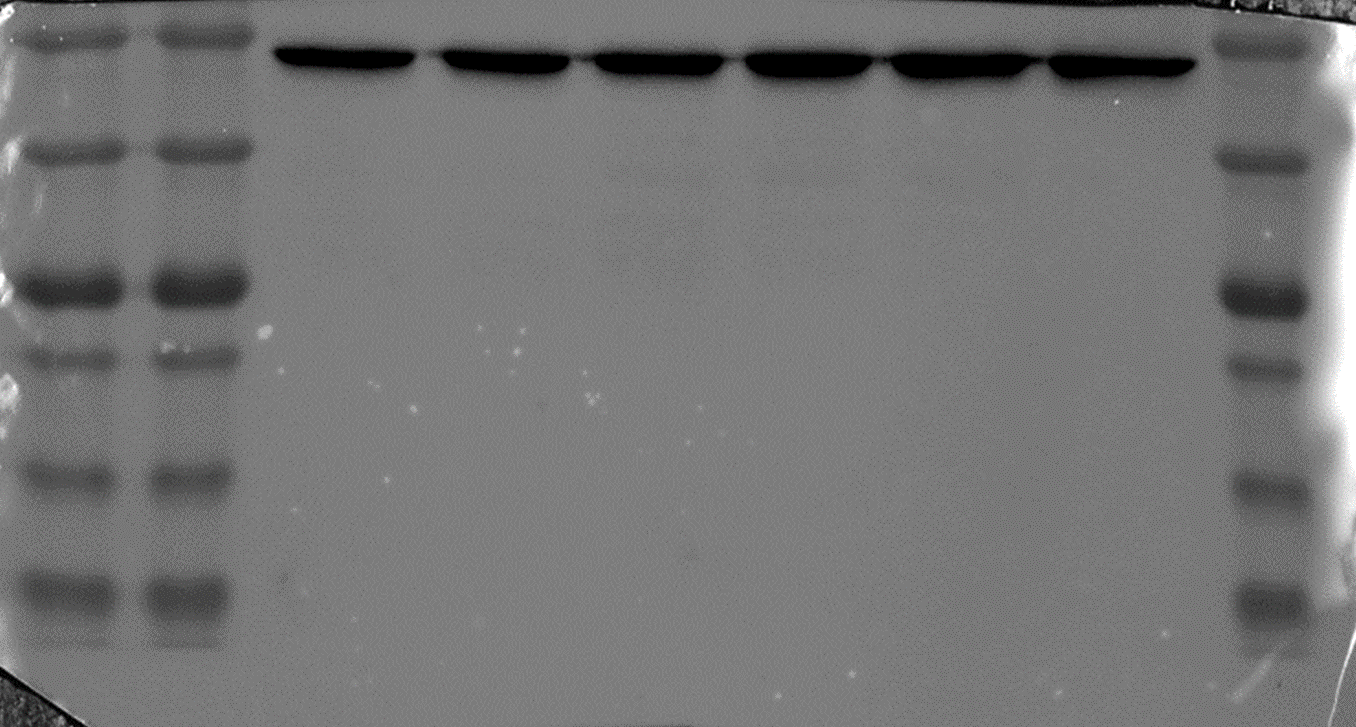


45 kDa

Original picture for **malignant Beta-actin** shown above





**(Malignant pFAK)**

**N1**

**N2**

**N3**

**T2**

**T1**

**T3**


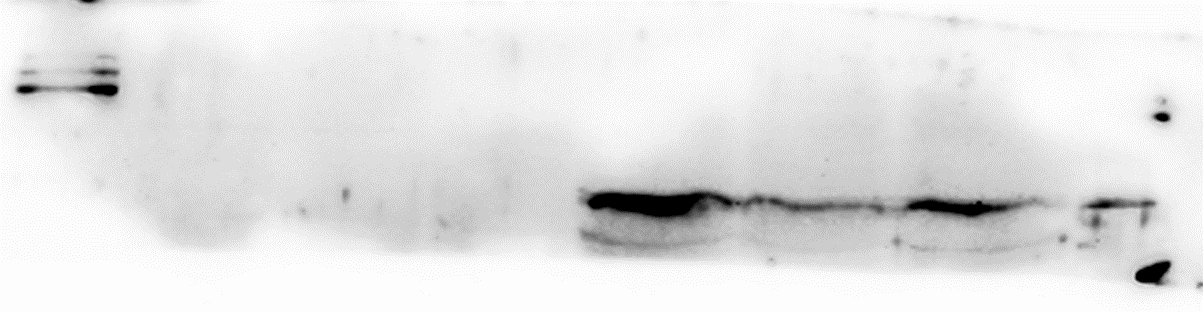


114 kDa

Original picture for **malignant pFAK** shown above


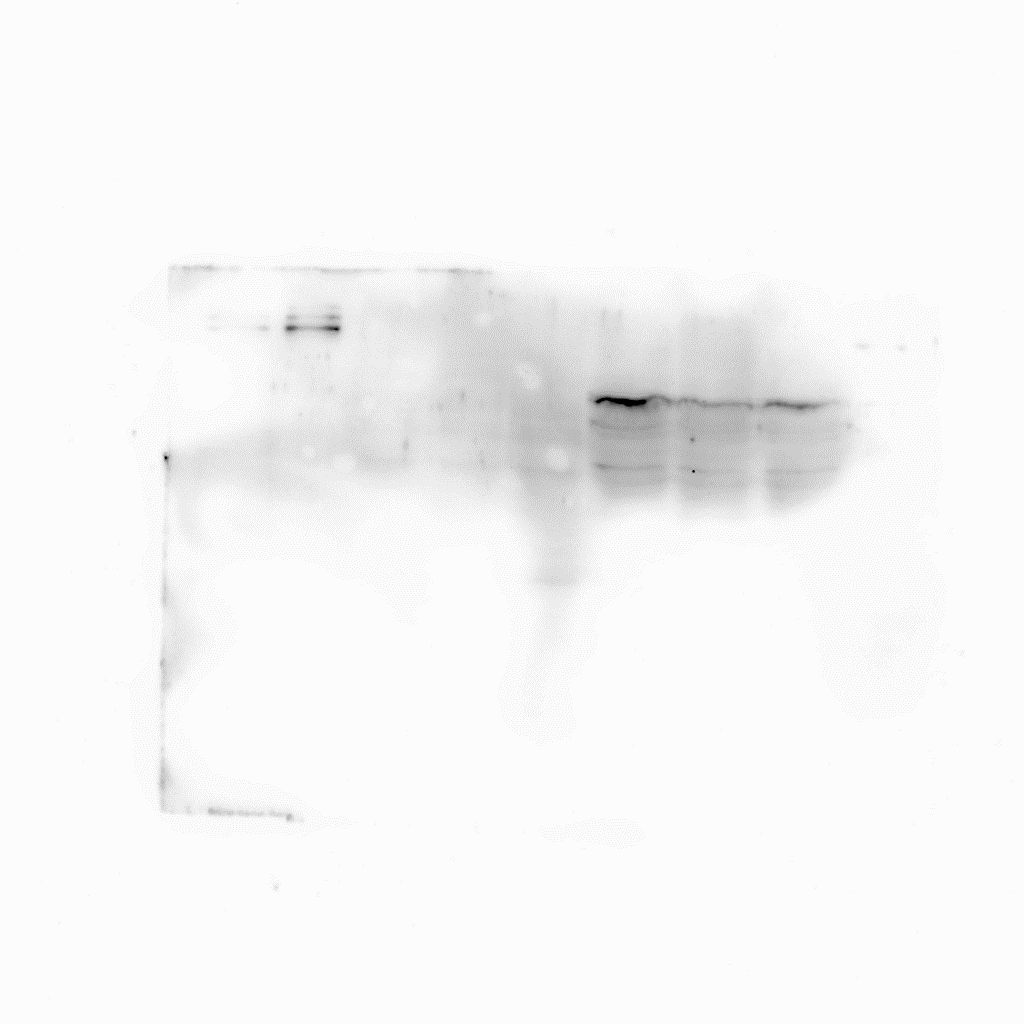


1. Beta-actin and pre-malignant RhoABC/ROCK1

**(Beta-actin)**


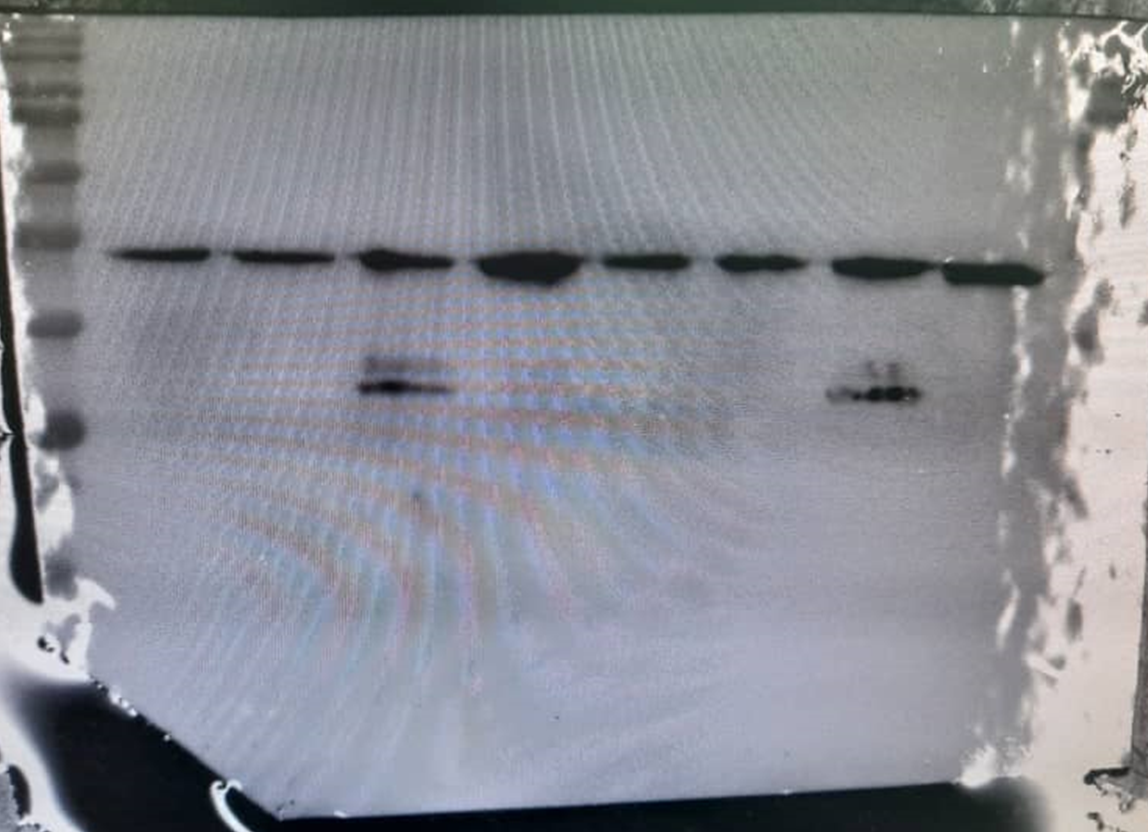


45 kDa

**T4**

**T3**

**N4**

**N3**

**T2**

**T1**

**N2**

**N1**

Original picture for **malignant Beta-actin** shown above


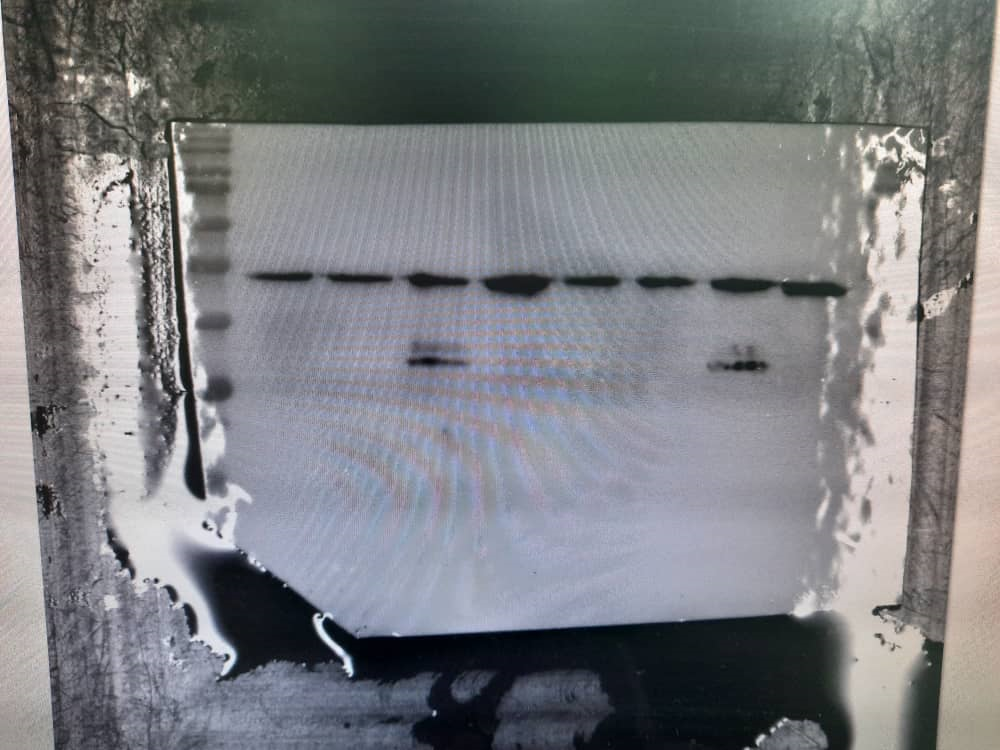


**(Pre-malignant RhoABC)**


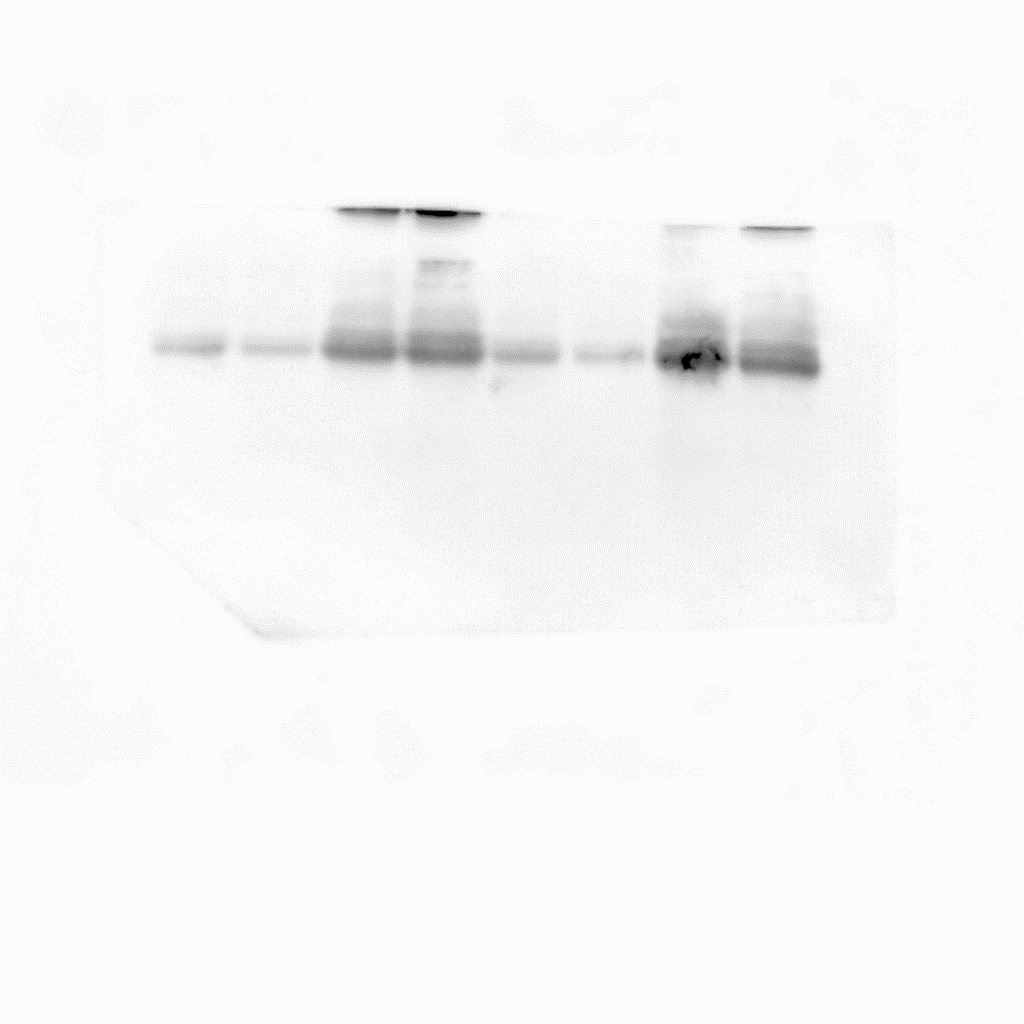


20 kDa

**N1**

**N2**

**T1**

**T2**

**N3**

**N4**

**T3**

**T4**

Above picture is already an original picture for **pre-malignant RhoABC**

**(Pre-malignant ROCK1)**

**T1**

**N2**

**T4**

**T3**

**N4**

**N3**

**T2**

**N1**


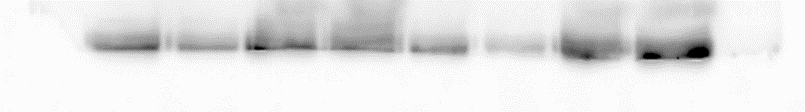


160 kDa

Original picture for **pre-malignant ROCK1** shown above


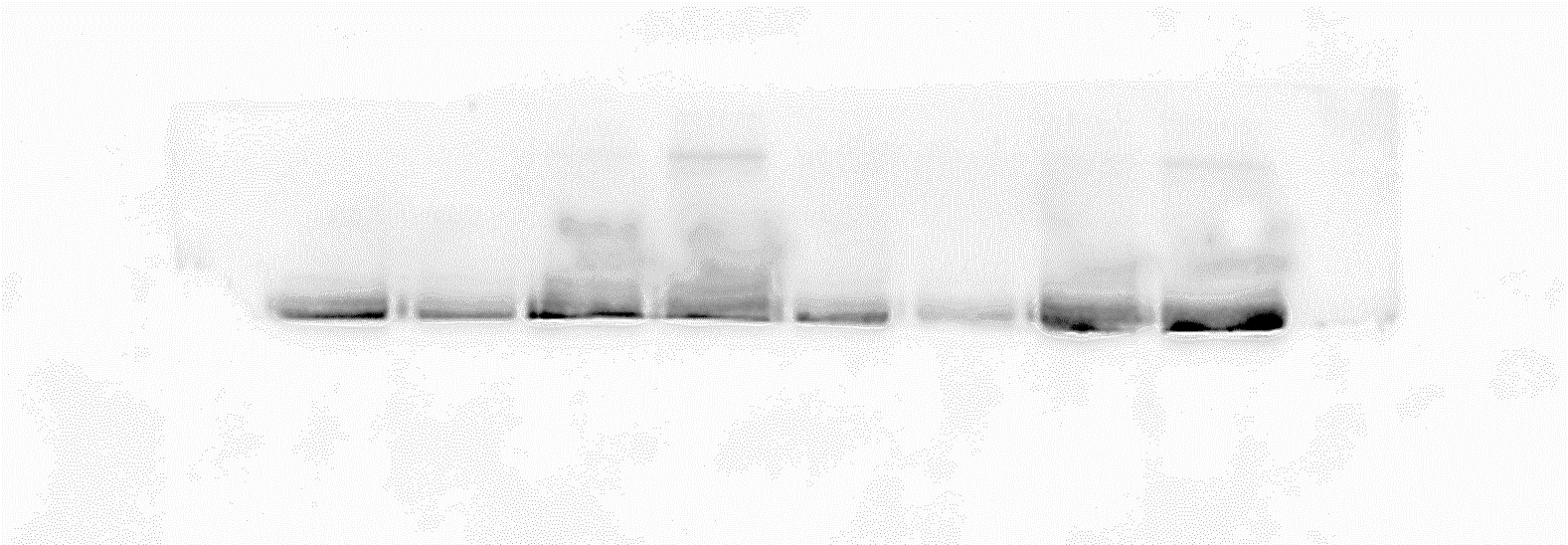


1. Beta-actin and malignant RhoABC/ROCK1

**(Beta-actin)**


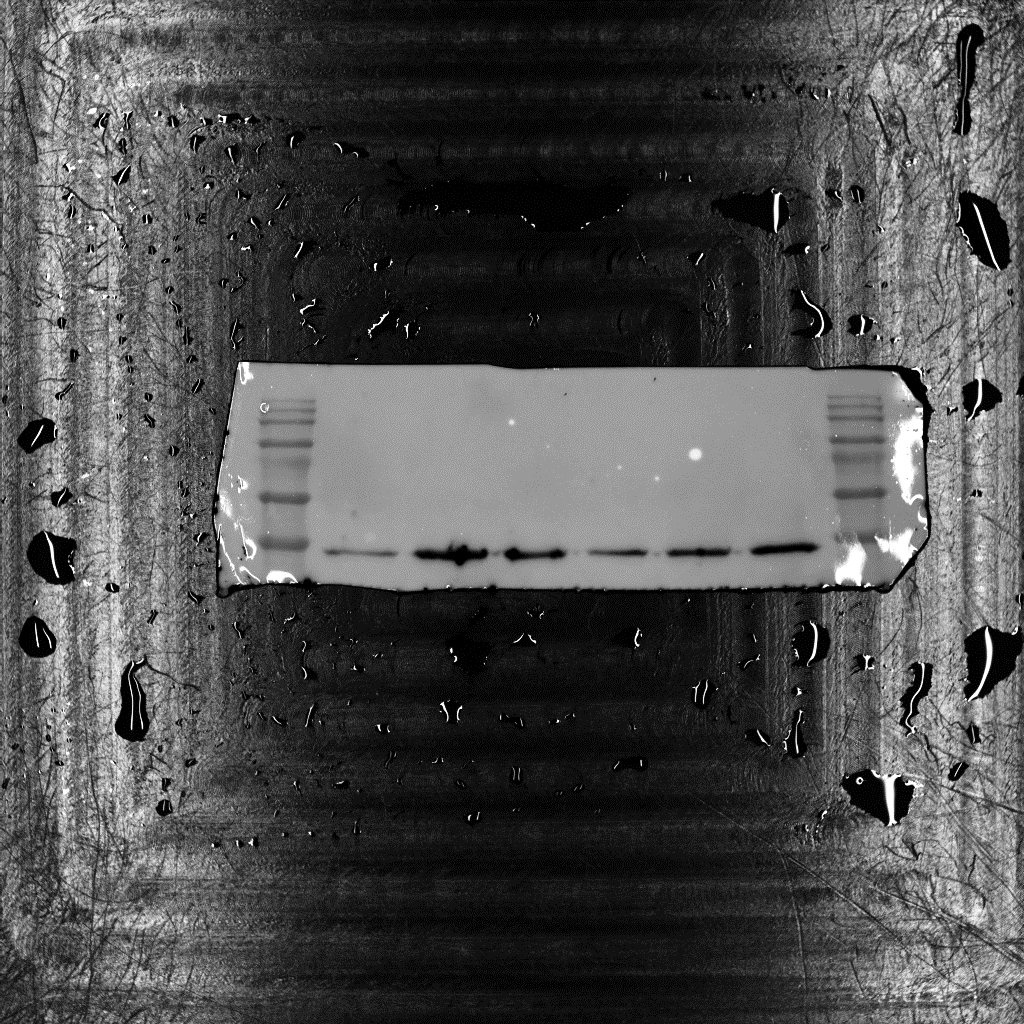


**T2**

**T1**

**N3**

**T3**

**N2**

**N1**

45 kDa

Above picture is already an original picture for **malignant Beta-actin**

**(Malignant RhoABC)**

**T3**

**T2**

**T1**

**N3**

**N2**


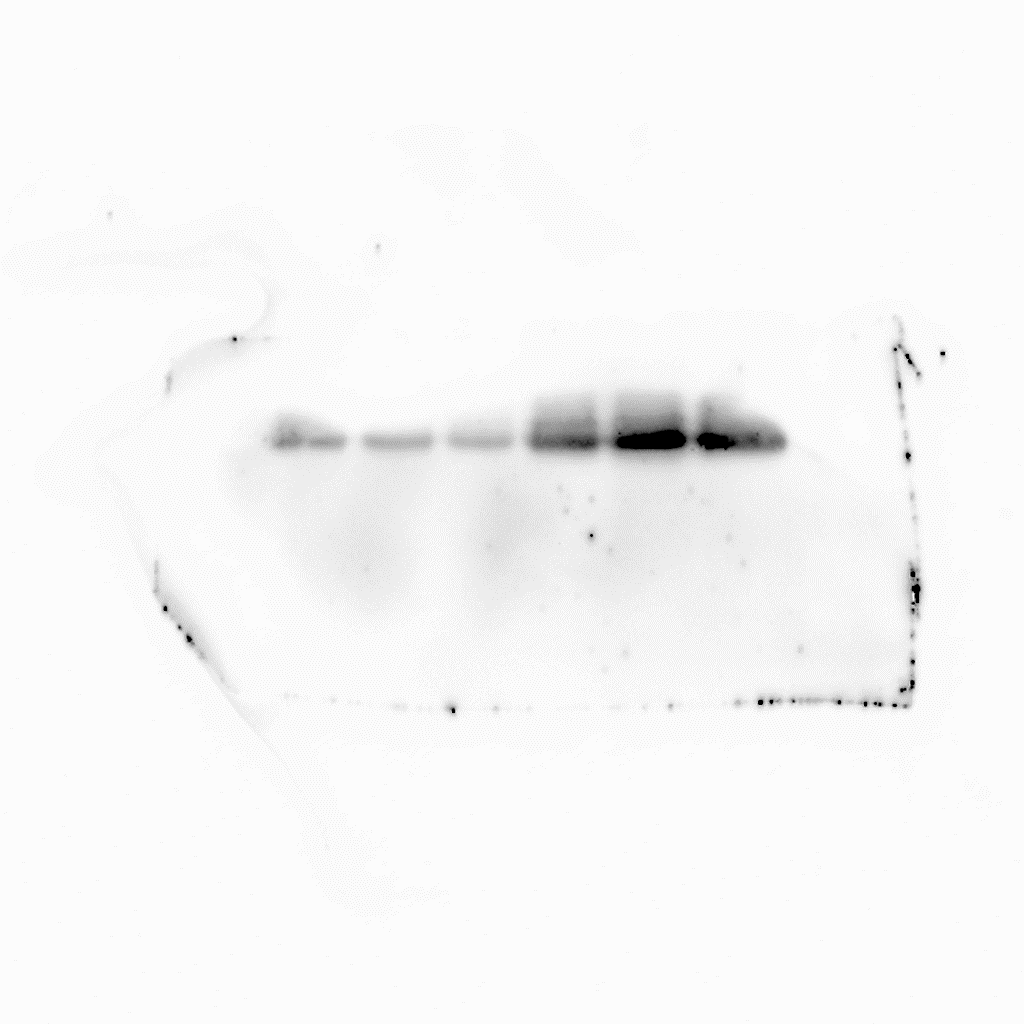


**N1**

20 kDa

Above picture is already an original picture for **malignant RhoABC**

**(Malignant ROCK1)**

**T1**

**T2**

**T3**

**N1**

**N2**

**N3**





160 kDa

Original picture for **malignant ROCK1** shown above





1. Beta-actin and pre-malignant ROCK2/pMLC

**(Beta-actin)**





**N3**

**N1**

**T1**

**T2**

**T3**

**N2**

45 kDa

Original article for **pre-malignant Beta-actin** shown above





**(Pre-malignant ROCK2)**

**T3**

**T2**

**T1**

**N3**

**N2**

**N1**


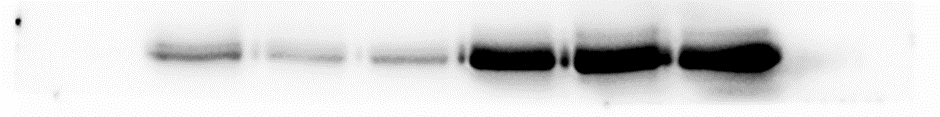


160 kDa

Original picture for **pre-malignant ROCK2** shown above


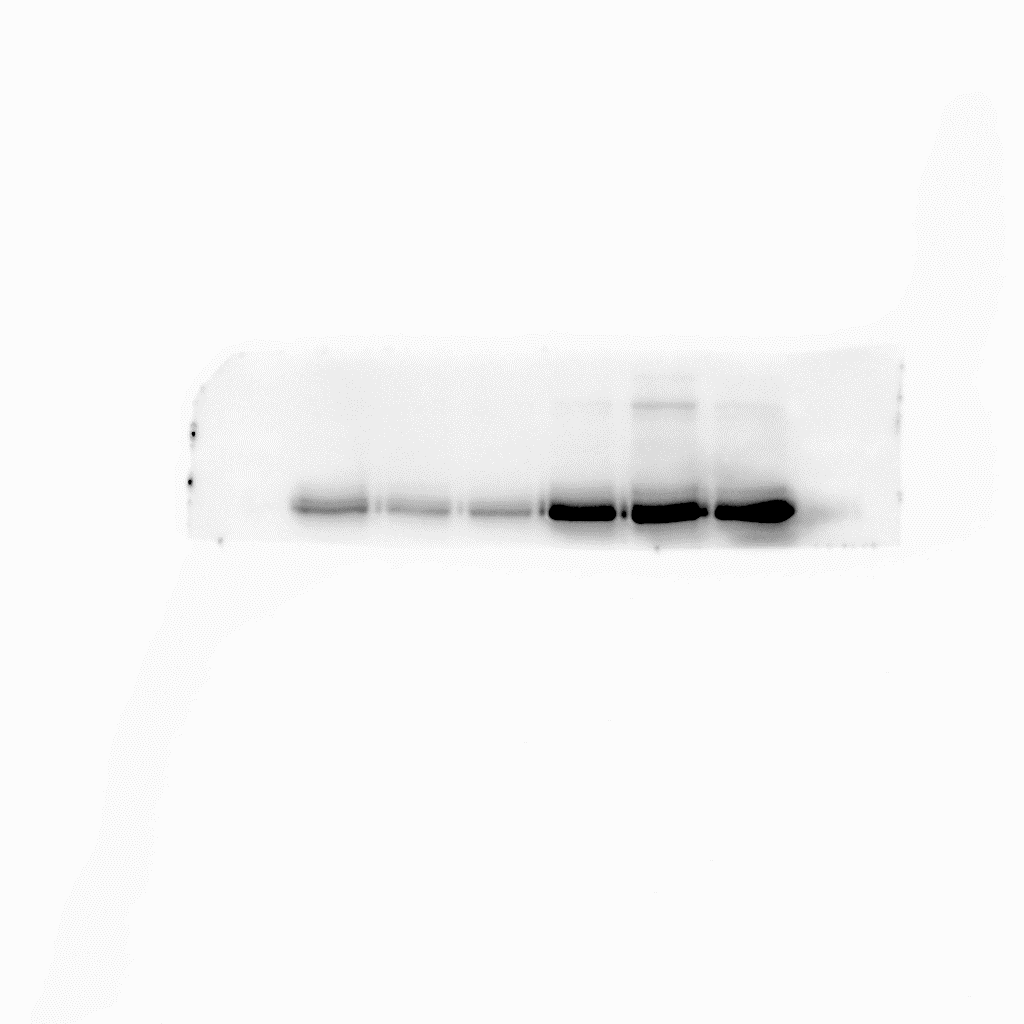


**(Pre-malignant pMLC)**


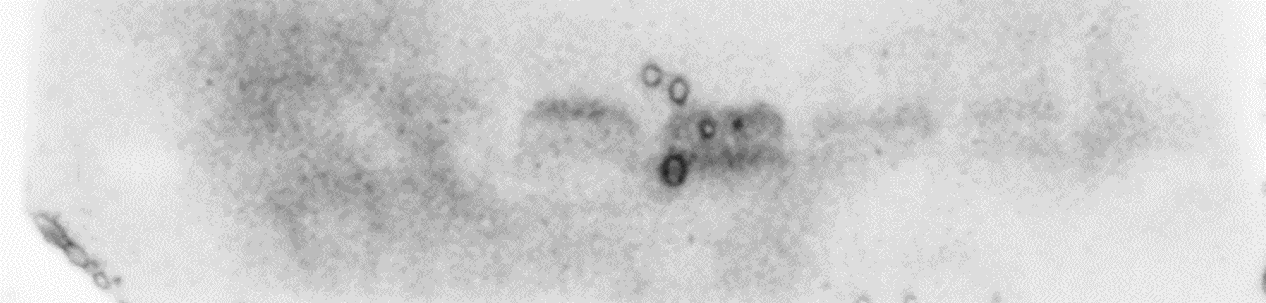


20 kDa

**N1**

**N2**

**N3**

**T1**

**T2**

**T3**

Original pictue for **pre-malignant pMLC** shown above


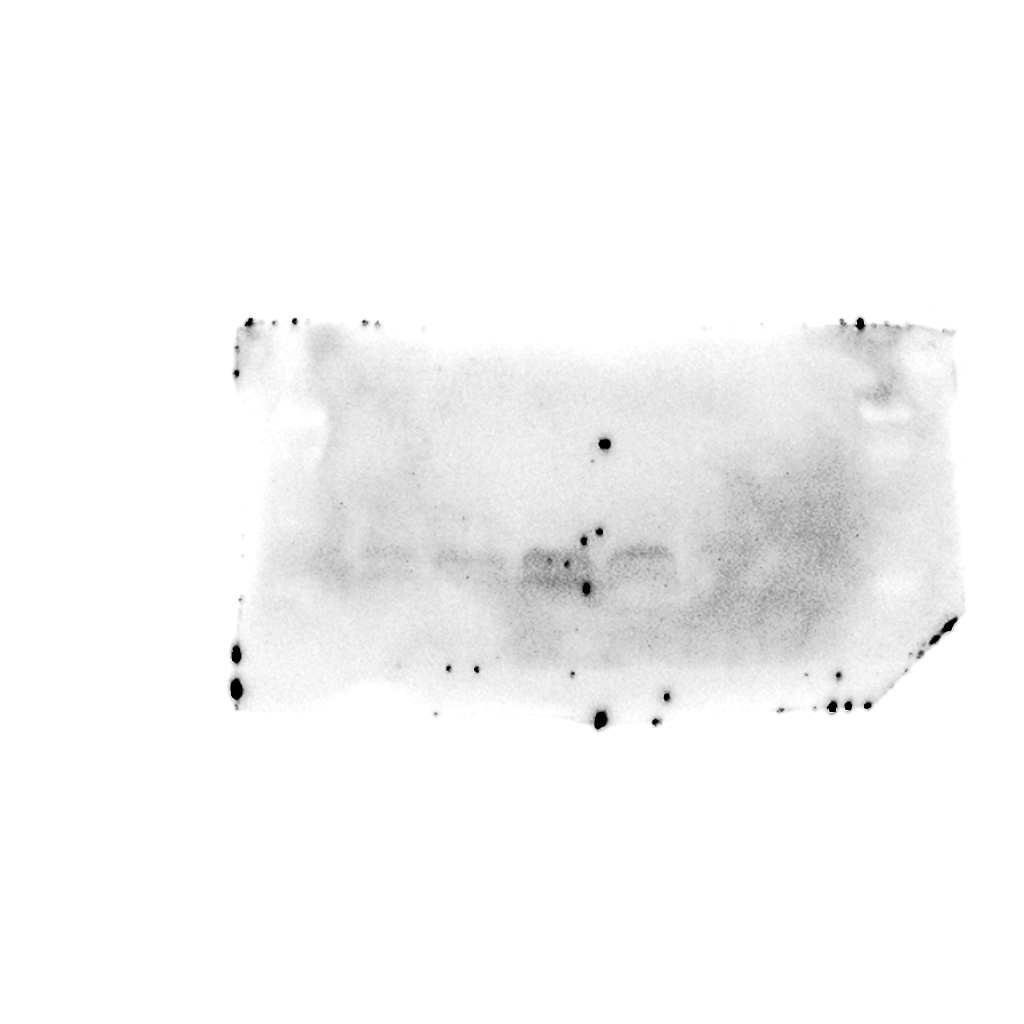


1. Beta-actin and malignant ROCK2/pMLC

**(Beta-actin)**


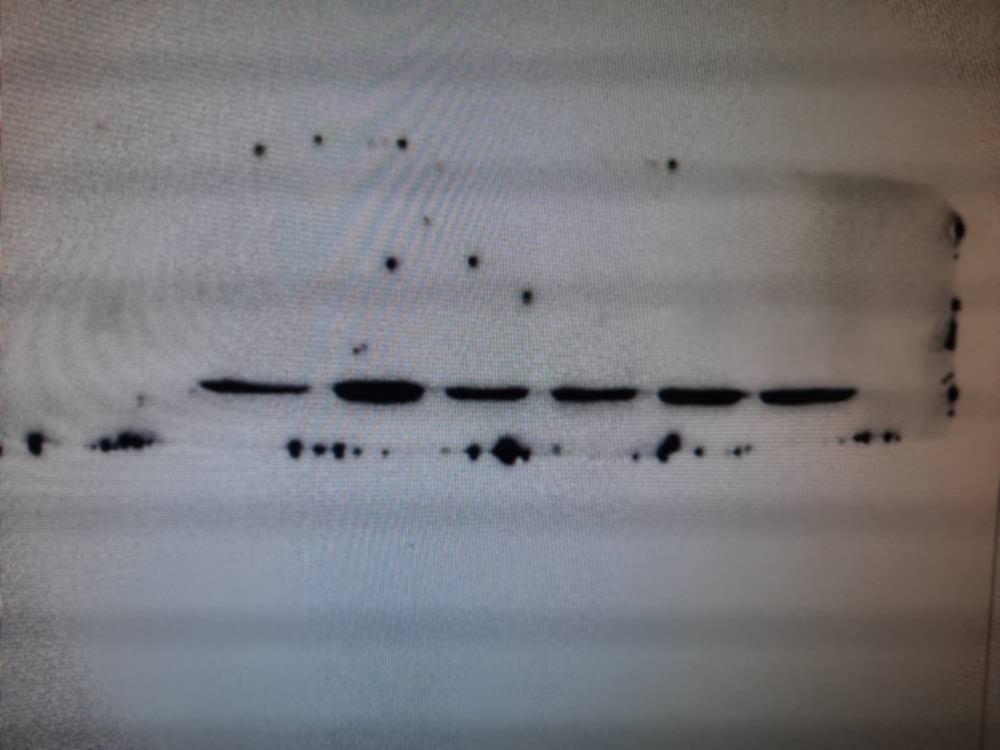


**T1**

**N1**

**T2**

**T3**

**N3**

**N2**

45 kDa

Original picture for **malignant Beta-actin** shown above


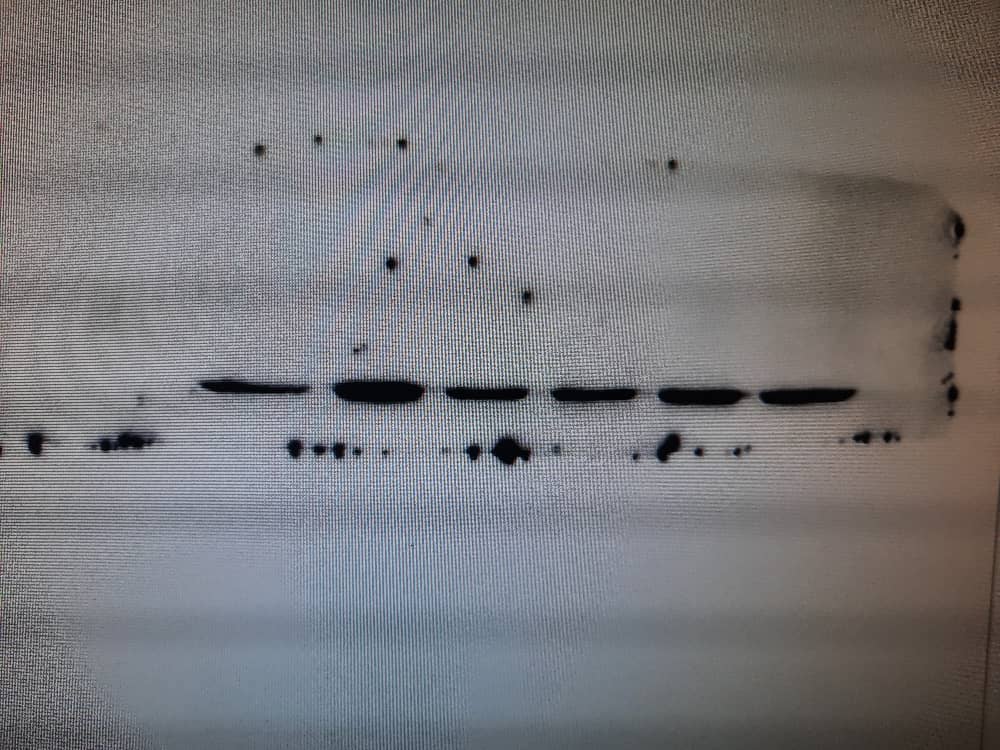


**(Malignant ROCK2)**

**N3**

**T1**

**T2**

**T3**

**N2**

**N1**


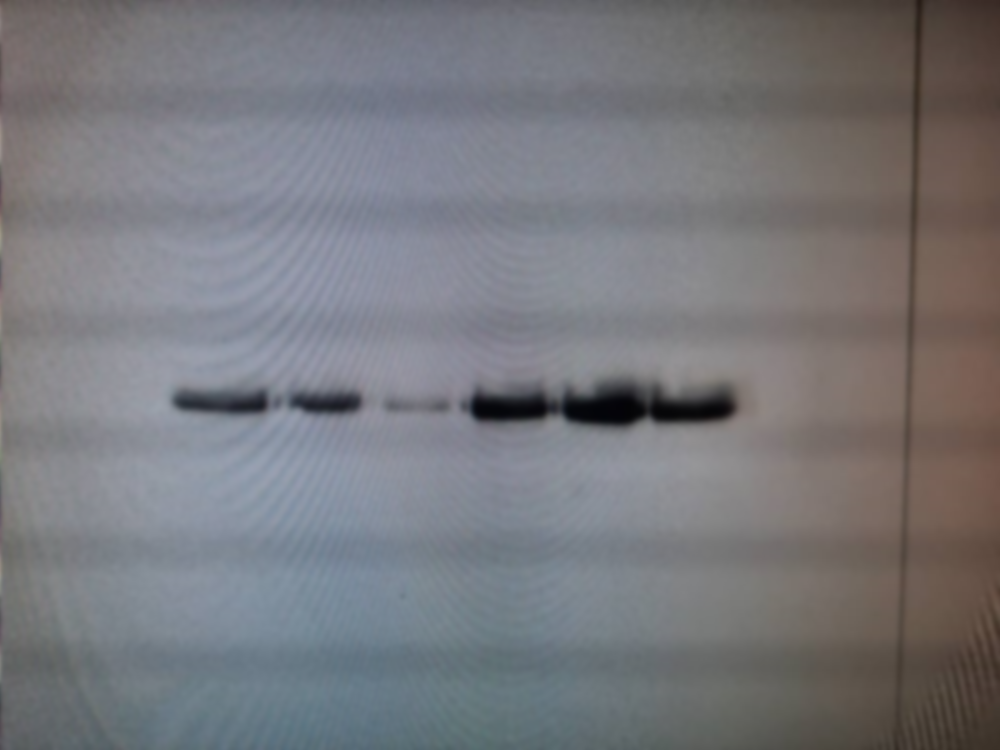


160 kDa

Original picture for **malignant ROCK2** shown above





**(Malignant pMLC)**

**T3**

**N1**

**N2**

**N3**

**T1**

**T2**


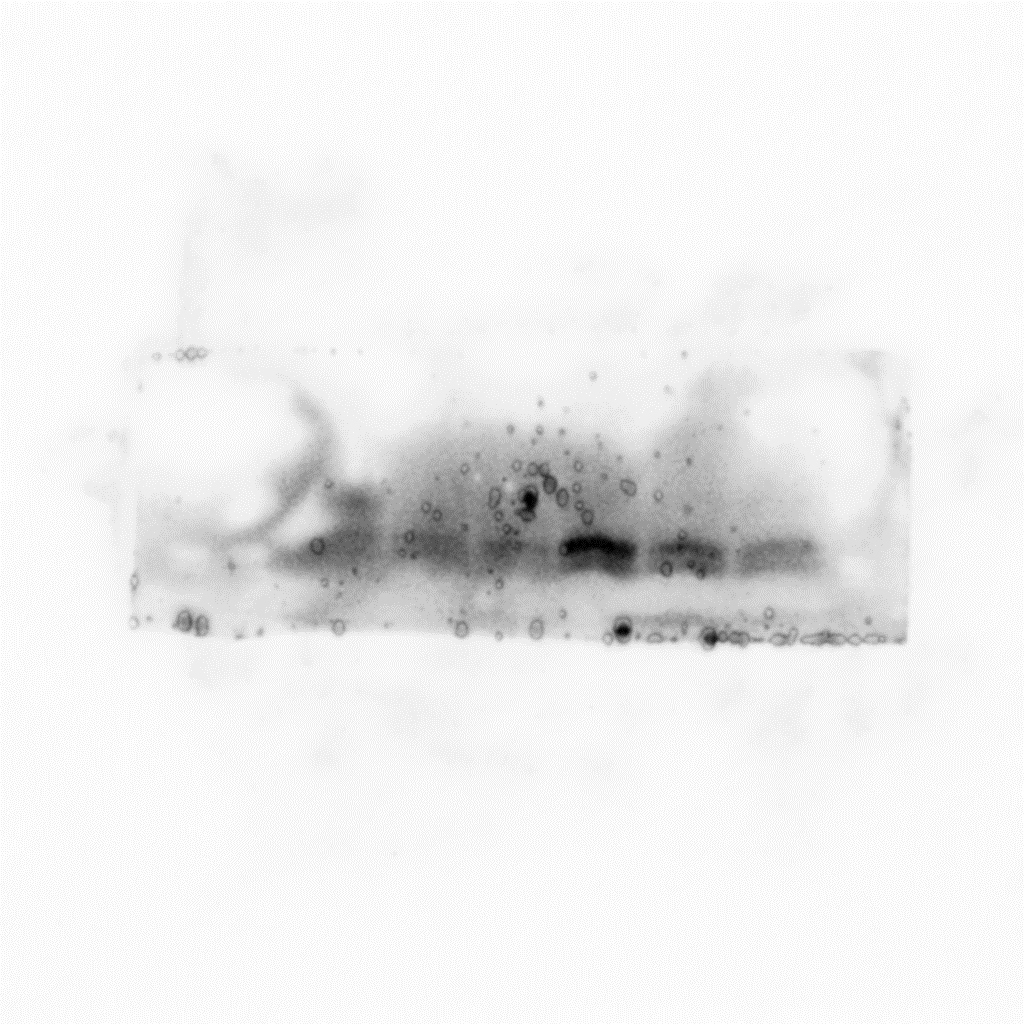


20 kDa

Original picture for **malignant pMLC** shown above
